# Comparative safety and effectiveness of oral anticoagulants in patients with non-valvular atrial fibrillation and high risk of gastrointestinal bleeding: A nationwide French cohort study

**DOI:** 10.1371/journal.pone.0310322

**Published:** 2024-11-15

**Authors:** Gregory Y. H. Lip, Robert Benamouzig, Anne-Céline Martin, Giancarlo Pesce, Gaelle Gusto, Nadia Quignot, Artak Khachatryan, Feng Dai, Fouad Sedjelmaci, Jose Chaves, Rupesh Subash, Ruth Mokgokong

**Affiliations:** 1 Liverpool Centre for Cardiovascular Science at University of Liverpool, Liverpool John Moores University and Liverpool Heart & Chest Hospital, Liverpool, United Kingdom; 2 Department of Clinical Medicine, Danish Center for Health Services Research, Aalborg University, Aalborg, Denmark; 3 Hôpital Avicenne, Bobigny, France; 4 European Hospital Georges Pompidou, Paris, France; 5 University of Paris, INSERM UMRS_1140, Paris, France; 6 Certara Italy, Milan, Italy; 7 Certara France, Paris, France; 8 Certara UK, London, United Kingdom; 9 Pfizer Inc., Groton, New York, United States of America; 10 Pfizer SAS, Paris, France; 11 Pfizer SLU., Madrid, Spain; 12 Pfizer LTD., Surrey, United Kingdom; Nanjing Drum Tower Hospital: Nanjing University Medical School Affiliated Nanjing Drum Tower Hospital, CHINA

## Abstract

**Background:**

This observational study compared effectiveness and safety of direct oral anticoagulants (DOACs; apixaban, rivaroxaban, dabigatran) or vitamin K antagonists (VKAs) in patients with non-valvular atrial fibrillation (NVAF) at high risk for gastrointestinal bleeding (GIB).

**Methods:**

Anticoagulant-naïve adults with NVAF with ≥1 GIB risk factor, initiating anticoagulant treatment January 2016–December 2019, and covered by the French national health data system were eligible. Outcomes included major bleeding (MB) and stroke/systemic embolism (SE). Patient characteristics were balanced using propensity score matching.

**Results:**

A total of 314,184 patients were identified with 162,150 (51.5%) in the apixaban cohort, 88,427 (28.1%) in the rivaroxaban cohort, 16,465 (5.2%) in the dabigatran cohort, and 47,142 (15.0%) in the VKA cohort (mean age 79.0 years, standard deviation 10.5; 51.0% female). A total of 45,124 apixaban-VKAs, 38,737 rivaroxaban-VKAs, 16,415 dabigatran-VKAs, 88,414 apixaban-rivaroxaban, 16,464 apixaban-dabigatran, and 16,459 rivaroxaban-dabigatran pairs were retained after propensity score matching. Apixaban had lower risk of MB versus dabigatran (hazard ratio [HR], 0.72; 95% confidence interval [CI], 0.63–0.83) and rivaroxaban (HR, 0.63; 95% CI, 0.59–0.66). Apixaban had lower risk of GIB versus dabigatran (HR, 0.46; 95% CI, 0.37–0.56) and rivaroxaban (HR, 0.54; 95% CI, 0.49–0.59). Risk of GIB was similar with dabigatran versus rivaroxaban (HR, 1.05; 95% CI, 0.89–1.24). Apixaban had lower risk of stroke/SE versus rivaroxaban (HR, 0.90; 95% CI, 0.84–0.96), while risk was similar versus dabigatran (HR, 1.1; 95% CI, 0.9–1.3). All DOACs had lower risk of MB and stroke/SE versus VKAs (*p*<0.001 for all).

**Conclusions:**

DOACs had improved safety and effectiveness from bleeding and stroke/SE, respectively, versus VKAs among patients with NVAF at high risk for GIB. Apixaban was associated with lower MB and GIB risk versus other DOACs. For stroke/SE, apixaban was associated with reduced risk versus rivaroxaban and similar risk versus dabigatran.

## Introduction

Atrial fibrillation (AF) is associated with a 4- to 5-fold increased risk of ischemic stroke, and stroke prevention with oral anticoagulants (OAC) is one of the pillars of AF management in guidelines [1]. The OACs include direct oral anticoagulants (DOACs), which are recommended by current guidelines as a first treatment option and are increasingly used for the prevention of stroke and/or systemic embolism (SE) in patients with non-valvular atrial fibrillation (NVAF) when compared to the vitamin K antagonists (VKAs) in real world clinical practice [2,3].

While the VKAs require routine coagulation monitoring, DOACs can be given in fixed doses without monitoring due to a larger therapeutic index. However, they may require dose adjustment based on specific patient characteristics such as age, renal function, and concomitant medications [4]. In a meta-analysis of the randomised controlled trials, the DOACs have a favorable risk-benefit profile versus VKAs, demonstrating reduced risk of stroke, intracranial hemorrhage (ICH), major bleeding (MB) and mortality [5]. However, an increased risk of gastrointestinal bleeding (GIB) was reported [5], which varies depending on the individual agent [6]; this is thought to be related to pre-existing gastrointestinal malignancies [7]. When compared with warfarin, real-world studies have also found a decreased risk of GIB with apixaban, a decreased-to-similar risk with dabigatran, and a similar-to-increased risk with rivaroxaban [6,8,9]. These previous studies assessed general populations of patients with NVAF and suggest that the risk of GIB can differ according to patients and the DOACs prescribed.

Current guidelines recommend that, in patients at high risk of GIB, a VKA or another DOAC preparation should be preferred over dabigatran 150 mg twice daily, rivaroxaban 20 mg once daily, or edoxaban 60 mg once daily [1,10]. Similarly, the CHEST guidelines recommend apixaban, edoxaban, or dabigatran 110 mg for patients with prior unprovoked bleeding, warfarin-associated bleeding, or those at high risk of bleeding, while those at high risk of GIB the use of apixaban or dabigatran 110 mg should be considered [11]. High risk factors for GIB include concomitant use of ulcerogenic agents, older age, renal impairment, *Helicobacter pylori* infection, and a history of GIB [12]. Identifying patients with NVAF at high risk for GIB based on these clinical risk factors is important, as variable patient characteristics must be considered when choosing an appropriate DOAC.

Our understanding of current risks and benefits of DOACs among patients with NVAF at high risk for GIB is limited, as patients prescribed DOACs in clinical practice may differ in age or comorbidities from those enrolled in clinical trials [13]. Understanding treatment patterns and sequencing for these patients, along with the effectiveness outcomes in real-world settings, represents an important challenge in NVAF and its management, particularly among those with high risk of GIB [14]. Hence, real-world evidence describing treatment patterns and effectiveness in this high-risk patient population would be valuable to clinicians and healthcare decision-makers.

The objective of this study was to describe and compare the use, safety, and effectiveness of OACs (apixaban, rivaroxaban, dabigatran, or VKAs) in patients with NVAF at high risk for GIB, utilizing recent data from a large French national healthcare database.

## Materials and methods

### Study design and data source

This was a historical, population-based cohort study (NCT05038228) using data extracted from the French national health data system (*Système National des Données de Santé*; SNDS) [15], which covers 99% of the French population. Data are linked via a unique social security number to primary care, hospital, pharmacy, and death registration databases, allowing for tracking of patient treatment history, treatment patterns, and hospitalizations based on International Classification of Diseases, Tenth Revision (ICD-10) codes. For this study, data spanning over a 6-year period from 1st January 2014 to 31st December 2019 (study period) was used. The study population included all anticoagulant (AC)-treatment naive adult patients (≥18 years old) diagnosed with NVAF and having a newly initiated AC treatment (index AC—apixaban, dabigatran, rivaroxaban or VKAs) during January 1, 2016 to December 31, 2019 (identification period). Index date was defined as the first AC prescription during the identification period. Follow-up was evaluated from the day after index date to censoring due to treatment switch, treatment discontinuation, treatment interruption, death, pregnancy, dialysis, chronic kidney disease stage V, or end of study. Treatment discontinuation was defined as >30 days after coverage by the last AC dispensation without refilling. Treatment interruption was defined as a patient having a gap with no new treatment within 30 days of the estimated end of supply, but subsequently restarts the index treatment after this period. Patients who had a reimbursement for an anticoagulant other than the index drug reimbursement during the follow-up period will be considered switchers. Baseline characteristics, comorbidities and prior anticoagulant use (to ensure population is AC-treatment naïve) were evaluated during the baseline period (during 24 months prior to and on the index date) using ICD-10 codes (principal or associated) and/or procedure and/or Anatomical Therapeutic Chemical (ATC) codes, as relevant. Concomitant treatments were identified using ATC codes and evaluated 3 months prior to and including the index date. Data were accessed for research purposes on 9 June 2022. Authors did not have access to information that could identify individual participants during or after data collection. The authors were authorized by the French national data protection authority (Commission Nationale de l’Informatique et des Libertés, CNIL) to access the data underlying this study (decision DR-2021-130).

### Study population

The study population included all AC-treatment naive adult patients (≥18 years old) diagnosed with NVAF, with high risk of GIB, and having a newly initiated AC treatment (index AC—apixaban, dabigatran, rivaroxaban or VKAs) during the identification period, i.e., from January 1, 2016 to December 31, 2019. Patients presenting ≥1 of the following indicators were considered as treated with AC for an indication of AF: (1) diagnoses identified through “Long Term Diseases” (LTD) registry in the 24 months before and the 30 days after index date using the ICD-10 code I48 (atrial fibrillation and flutter); (2) main or associated diagnoses of hospitalizations in the 24 months before index date using the ICD-10 code I48; and (3) prescription of any anti-arrhythmic drugs (ATC: C01B*) dispensed concomitantly with VKAs or DOACs, i.e., dispensed between 6 weeks before and 15 days after index date (probable AF patients).

Patients at high risk of GIB were identified as follows: (1) older age (≥75 years) at index date [12,16]; (2) impaired kidney function (chronic kidney disease, stage III-IV) during the baseline period of 24 months [12,17]; (3) a score of 3 or greater on the HAS-BLED (hypertension, kidney or liver disease, stroke history, prior bleeding, unstable international normalized ratio, age >65, drug or alcohol use) [12]; (4) concomitant use of NSAIDs, antiplatelet agents, or corticosteroids within 6 weeks prior to index date [12,17]; and (5) a past history of GI ulcers or bleeds (i.e., peptic ulcer, prior GIB, *Helicobacter pylori* infection, diverticulosis, angiodysplasias, GI cancer, hereditary hemorrhagic disease, or other GI lesion) during the baseline period of 24 months [12,18]. The population included patients who met selection criteria and received treatment with apixaban, dabigatran, rivaroxaban, or VKAs after their initial index encounter. VKAs included warfarin, acenocoumarol, or fluindione; of note, NVAF patients who received edoxaban were not included as it was not available in France.

**Fig 1:**
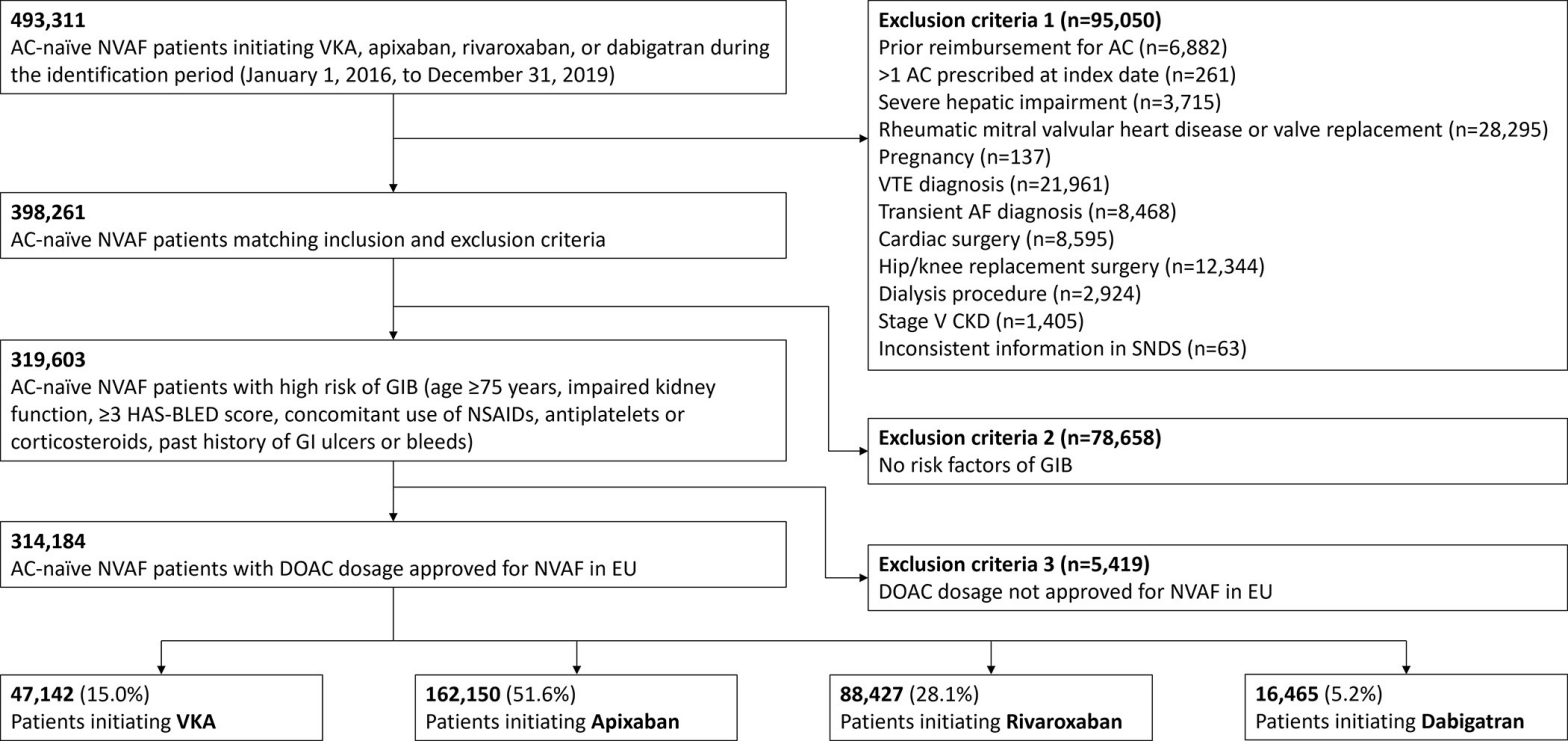
Patient selection criteria.

Patients were excluded if they had: (1) ≥1 reimbursement for an AC treatment within 24 months prior to the index date; (2) patient prescribed with mixed types of AC treatments at the index date; (3) mixed DOAC dosage at index date (e.g., apixaban 5 mg + 2.5 mg) or DOAC dosage not approved for NVAF in the European Union (e.g., rivaroxaban 10 mg or dabigatran 75 mg); (4) severe hepatic impairment within 24 months prior to the index date; (5) rheumatic mitral valvular heart disease or a valve replacement procedure within 24 months prior to the index date; or (6) evidence of pregnancy in the 9 months prior to the index date. Full exclusion criteria are listed in **Fig 1**.

### Outcome measures

The primary safety outcomes included MB leading to hospitalization, overall and by site (GIB, ICH, and other). The primary effectiveness outcomes included stroke (ischemic or hemorrhagic) or SE, overall and separately; ICD-10 codes used to identify these outcomes are presented in **S1 Table**. Both effectiveness and safety outcomes were identified with primary diagnosis and evaluated from the day after the index date until end of the follow-up period.

### Statistical analysis

A 1:1 propensity score (PS) matching technique utilizing binary logistic regression was used as the primary method to balance patient characteristics between the cohorts. PS was defined as the probability of a patient receiving a certain treatment conditional on their observed baseline covariates. Covariates key demographic characteristics (including sociodemographic characteristics), comorbidities and baseline concomitant medication use. PS matching for six different groups was performed: apixaban versus VKAs (as a group), apixaban versus dabigatran, apixaban versus rivaroxaban, rivaroxaban versus dabigatran, rivaroxaban versus VKAs (as a group), and dabigatran versus VKAs (as a group). Two patients from two cohorts were matched using a sequential pairwise nearest neighbor approach, if the difference in the logit of PS between them was equal to 0.2 times the standard deviation of the logit of the PS. Several checks were performed to ensure a good balance of PS and of covariates between treatment groups. First, the treatment group PS distribution was analyzed graphically using a love plot. Second, the balance of covariates across treatment and comparison groups was checked using the absolute standardized difference (with a threshold of <10%). Descriptive statistics were provided for baseline demographic and clinical characteristics for both the pre- and post-PS-matched between-group comparisons, overall and by index AC treatment.

The time-to-event of first occurrence of each outcome of interest and cumulative incidence curves were estimated using Kaplan-Meier methodology after PS matching. To compare risk, the incidence rates for clinical outcomes (including 95% confidence interval [CI] within each cohort), censored at treatment non-persistence, chronic kidney disease stage V, dialysis, pregnancy, death, or end of the study, were calculated as the number of patients who experienced the event divided by the observed time at risk expressed per 100 person-years. The hazard ratios (HR) and associated 95% CI were calculated for each studied event (MB [overall, GI, ICH, other] and stroke/SE [overall, hemorrhagic, ischemic, SE]) using Cox proportional hazard models. Proportional hazard assumption was verified visually and using Schoenfeld residuals plots. Statistical significance was set at *p*<0.05, and all tests were 2-tailed. All analyses were conducted using R (version 4.1.3) or SAS Statistical Package (SAS guide 8).

#### Subgroup analyses

Descriptive and comparative analyses of outcomes adjusted on PS were conducted for patients initiating standard dosing (apixaban 5 mg, rivaroxaban 20 mg, dabigatran 150 mg) and reduced dosing (apixaban 2.5 mg, rivaroxaban 15 mg, dabigatran 110 mg) in each treatment cohort. For each comparison, the PS was performed and evaluated to ensure a good balance of covariates between the groups compared. For VKA–DOAC comparisons, PS matchings were newly performed; as standard/reduced dosage information was not available for VKAs, patients initiating VKAs were matched with both standard and reduced dosage DOAC subgroups. For DOAC–DOAC comparisons, as index DOAC dosage was already included in the PS matching and the standard and reduced dose subgroups were still balanced, data were analyzed without performing a new PS matching.

### Sensitivity analyses

Sensitivity analyses of comparative safety and effectiveness were also conducted using accelerated failure time (AFT) models to compare results with the Cox model due to proportional hazards violations [19]. Gamma, Weibull, exponential, log normal, and log logistic distributions were considered to model the data; the distribution with the lowest Akaike information criterion was retained. Additional sensitivity analyses included evaluating VKA versus DOACs and DOAC versus DOAC, excluding patients identified using anti-arrhythmic drug prescriptions without a diagnosis code for AF (probable AF patients) from the PS-matched cohorts, adjusting for imbalanced covariate distributions using Cox models (**S2 and S3 Tables**).

## Results

### Patient selection and characteristics

A total of 493,311 AC-naïve patients initiating treatment were identified in the SNDS between January 2016 and December 2019. After applying the selection criteria, a total of 314,184 patients were included in the final study population of patients with NVAF at high risk of GIB, with 162,150 in the apixaban cohort, 88,427 in the rivaroxaban cohort, 16,465 in the dabigatran cohort, and 47,142 in the VKA cohort (**Fig 1**). Of these, 231,075 (73.5%) patients had confirmed NVAF and 83,109 (26.5%) had probable NVAF.

Prior to PS matching, overall mean age at index date was 79.0 (standard deviation [SD] 10.5) years; 51.0% were female, and mean Charlson Comorbidity Index score was 1.8 (SD 2.0; **S4 Table**). Overall mean CHA_2_DS_2_-VASc score was 3.9 (SD 1.5). Overall, 57.2% of DOAC recipients initiated on standard dosage, whereas the remaining 42.8% initiated DOAC treatment on reduced dose (**S4 Table)**.

Among the identified high-risk patients, a total of 226,037 (71.9%) patients were aged ≥75 years, 186,023 (59.2%) had a HAS-BLED score ≥3, 193,868 (61.7%) were receiving medications associated with GI bleeding risk (antiplatelets, NSAIDs or corticosteroids), 18,703 (6.0%) had renal impairment (chronic kidney disease, stage III-IV), and 25,200 (8.0%) had a prior GI condition.

### Risk of bleeding and stroke/SE

#### Incidence rates prior to PS matching

Overall, 10,702 patients (3.4%) had a MB event; of these, more than one-third was hospitalized due to GIB (n = 4,117, 1.3%). A total of 6,467 (2.1%) patients had a hospitalization due to stroke or SE. Prior to PS matching, patients in the VKA cohort had an incidence rate of 6.1 (95% CI, 5.9–6.4) events per 100 person-year for MB leading to hospitalization, whereas patients in the apixaban, rivaroxaban, and dabigatran cohorts had incidence rates of 2.5 (95% CI, 2.4–2.5), 3.4 (95% CI, 3.3–3.6), and 2.7 (95% CI, 2.5–3.0) events per 100 person-year, respectively (**S5 Table**).

#### PS matched cohort

A total of 45,124 (14.4% of total study population) apixaban-VKAs, 38,737 (12.3%) rivaroxaban-VKAs, 16,415 (5.2%) dabigatran-VKAs, 88,414 (28.1%) apixaban-rivaroxaban, 16,464 (5.2%) apixaban-dabigatran, and 16,459 (5.2%) rivaroxaban-dabigatran pairs were retained after PS matching. Demographic and clinical characteristics were similar (i.e., absolute standardized difference <10%) for the PS-matched cohorts in the DOACs versus VKAs comparison (**Table 1**) and in the DOACs versus DOACs comparison (**Table 2**).

**Table 1:** Baseline characteristics of patients prescribed DOACs versus VKAs after PS matching.

Characteristic, n (%)		VKAs versus apixaban			VKAs versus rivaroxaban			VKAs versus dabigatran		
		VKAs (n = 45,124)	Apixaban (n = 45,124)	Standardized difference	VKAs (n = 38,737)	Rivaroxaban (n = 38,737)	Standardized difference	VKAs (n = 16,415)	Dabigatran (n = 16,415)	Standardized difference
**Atrial fibrillation identification setting**	Inpatient claim with I48 code	34800 (77.12%)	35035 (77.64%)	1.24	28743 (74.2%)	28984 (74.82%)	1.43	9293 (56.61%)	9250 (56.35%)	-0.53
	LTR registration with I48 code*	2052 (4.55%)	2087 (4.63%)	0.37	2033 (5.25%)	2180 (5.63%)	1.67	1684 (10.26%)	1887 (11.5%)	3.97
	Use of anti-arrhythmic drugs*	8272 (18.33%)	8002 (17.73%)	-1.56	7961 (20.55%)	7573 (19.55%)	-2.5	5438 (33.13%)	5278 (32.15%)	-2.08
**Follow up time (months)** ^†^	Mean (SD)	10.9 (11.6)	13 (12.2)		11.1 (11.7)	12.7 (12.4)		11.0 (12.0)	13.5 (12.7)	
	Median (IQR)	6.1 (2.3–15.9)	8.9 (3.1–20.2)		6.2 (2.3–16.2)	8.1 (2.7–20.2)		5.9 (2.0–16.4)	9.0 (2.6–22.7)	
**Age at index date (years), mean (SD)**		81.4 [10.2]	80.5 [10.3]	-8.27	80.6 [10.3]	79.5 [10.7]	-9.79	78.3 [10.1]	77.8 [10.1]	-4.49
**Age groups at index date**	18–54 years	782 (1.73%)	863 (1.91%)	1.34	765 (1.97%)	1025 (2.65%)	4.47	399 (2.43%)	392 (2.39%)	-0.28
	55–64 years*	2332 (5.17%)	2705 (5.99%)	3.6	2225 (5.74%)	2626 (6.78%)	4.27	1155 (7.04%)	1252 (7.63%)	2.27
	65–74 years*	6681 (14.81%)	7549 (16.73%)	5.28	6198 (16%)	6768 (17.47%)	3.94	3227 (19.66%)	3493 (21.28%)	4.02
	75–79 years*	6087 (13.49%)	6557 (14.53%)	3	5672 (14.64%)	5822 (15.03%)	1.09	3175 (19.34%)	3175 (19.34%)	0
	80–84 years*	9111 (20.19%)	8893 (19.71%)	-1.21	8039 (20.75%)	7833 (20.22%)	-1.32	3812 (23.22%)	3630 (22.11%)	-2.65
	85–89 years*	10520 (23.31%)	10053 (22.28%)	-2.47	8752 (22.59%)	8361 (21.58%)	-2.43	3081 (18.77%)	2935 (17.88%)	-2.30
	≥90 years*‡	9611 (21.3%)	8504 (18.85%)	-6.13	-	-	-	-	-	-
	90–94 years*	-	-	-	5529 (14.27%)	4973 (12.84%)	-4.19	1274 (7.76%)	1258 (7.66%)	-0.37
	≥95 years*	-	-	-	1557 (4.02%)	1329 (3.43%)	-3.11	292 (1.78%)	280 (1.71%)	-0.56
**Sex**	Male	21532 (47.7%)	22015 (48.8%)		18749 (48.4%)	19470 (50.3%)		8314 (50.65%)	8443 (51.4%)	
	Female*	23592 (52.28%)	23109 (51.21%)	2.14	19988 (51.6%)	19267 (49.74%)	3.72	8101 (49.35%)	7972 (48.57%)	1.57
**GIB risk factors**	Age ≥75 years	35329 (78.29%)	34007 (75.36%)	-6.95	29549 (76.28%)	28318 (73.1%)	-7.31	11634 (70.87%)	11278 (68.71%)	-4.72
	HAS-BLED score, mean	3.1 [1.1]	3.1 [1.1]		2.9 [1]	2.9 [1]		2.6 [1]	2.6 [1]	
	0	108 (0.24%)	170 (0.38%)	2.48	108 (0.28%)	197 (0.51%)	3.67	95 (0.58%)	112 (0.68%)	1.31
	1	2486 (5.51%)	2390 (5.3%)	-0.94	2485 (6.42%)	2586 (6.68%)	1.05	1677 (10.22%)	1749 (10.65%)	1.43
	2	10834 (24.01%)	10198 (22.6%)	-3.33	10683 (27.58%)	10545 (27.22%)	-0.8	5289 (32.22%)	5232 (31.87%)	-0.74
	≥3*	31696 (70.24%)	32366 (71.73%)	3.27	25461 (65.73%)	25409 (65.59%)	-0.28	9354 (56.98%)	9322 (56.79%)	-0.39
	Concomitant medications	26197 (58.06%)	28303 (62.72%)	9.55	22717 (58.64%)	24255 (62.61%)	8.13	9862 (60.08%)	10098 (61.52%)	2.95
	Renal impairment	6287 (13.93%)	5593 (12.39%)	-4.55	3153 (8.14%)	2589 (6.68%)	-5.56	458 (2.79%)	395 (2.41%)	-2.41
	Prior GI condition	3990 (8.84%)	4664 (10.34%)	5.07	3262 (8.42%)	3674 (9.48%)	3.73	1319 (8.04%)	1368 (8.33%)	1.09
**Number of GIB risk factors**	1	12529 (27.77%)	11229 (24.88%)	-6.55	12341 (31.86%)	11910 (30.75%)	-2.4	6397 (38.97%)	6476 (39.45)	0.99
	2	11398 (25.26%)	12553 (27.82%)	5.8	9967 (25.73%)	10536 (27.2%)	3.33	4375 (26.65%)	4413 (26.88%)	0.52
	3	16885 (37.42%)	17057 (37.8%)	0.79	13965 (36.05%)	14030 (36.22%)	0.35	5104 (31.09%)	4970 (30.28%)	-1.77
	4	4041 (8.96%)	3998 (8.86%)	-0.33	2348 (6.06%)	2132 (5.5%)	-2.39	527 (3.21%)	531 (3.23%)	0.14
	5	271 (0.6%)	287 (0.64%)	0.45	116 (0.3%)	129 (0.33%)	0.6	12 (0.07%)	25 (0.15%)	2.36
**Charlson Comorbidity Index score**	0	7612 (16.87%)	6652 (14.74%)	-5.83	7579 (19.57%)	6955 (17.95%)	-4.13	5486 (33.42%)	5739 (34.96%)	3.25
	1 or 2	18312 (40.58%)	18971 (42.04%)	2.97	17447 (45.04%)	18514 (47.79%)	5.53	7113 (43.33%)	6961 (42.41%)	-1.87
	3 or 4	11668 (25.86%)	11879 (26.33%)	1.06	8984 (23.19%)	8644 (22.31%)	-2.09	2663 (16.22%)	2558 (15.58%)	-1.75
	≥5	7532 (16.69%)	7622 (16.89%)	0.53	4727 (12.20%)	4624 (11.94%)	-0.82	1153 (7.02%)	1157 (7.05%)	0.1
**Additional comorbidities**	Myocardial infarction*	5053 (11.2%)	5055 (11.2%)	0.01	4015 (10.36%)	4114 (10.62%)	0.83	909 (5.54%)	886 (5.4%)	-0.56
	Congestive heart failure*	20526 (45.49%)	19549 (43.32%)	-4.36	15816 (40.83%)	15444 (39.87%)	-1.96	3553 (21.64%)	3622 (22.07%)	1.02
	Peripheral vascular disease*	5912 (13.1%)	6119 (13.56%)	1.35	4462 (11.52%)	4515 (11.66%)	0.43	1098 (6.69%)	1110 (6.76%)	0.29
	Cerebrovascular disease*	8255 (18.29%)	9540 (21.14%)	7.16	6609 (17.06%)	6792 (17.53%)	1.25	3095 (18.85%)	3137 (19.11%)	0.65
	Dementia*	4997 (11.07%)	5190 (11.5%)	1.35	3930 (10.15%)	3866 (9.98%)	-0.55	863 (5.26%)	847 (5.16%)	-0.44
	Chronic pulmonary disease*	10239 (22.69%)	11566 (25.63%)	6.87	8643 (22.31%)	9560 (24.68%)	5.59	3157 (19.25%)	3130 (19.07%)	-0.42
	Connective tissue disease*	820 (1.82%)	1015 (2.25%)	3.06	667 (1.72%)	729 (1.88%)	1.2	211 (1.29%)	206 (1.25%)	-0.27
	Ulcer disease*	702 (1.56%)	716 (1.59%)	0.25	508 (1.31%)	493 (1.27%)	-0.34	168 (1.02%)	158 (0.96%)	-0.61
	Mild liver disease*	1188 (2.63%)	1202 (2.66%)	0.19	974 (2.51%)	930 (2.4%)	-0.73	241 (1.47%)	218 (1.33%)	-1.19
	Diabetes*	10956 (24.28%)	11343 (25.14%)	1.99	8902 (22.98%)	9172 (23.68%)	1.65	3160 (19.25%)	3074 (18.73%)	-1.34
	Diabetes with end-organ damage*	1984 (4.4%)	1803 (4%)	-2	1191 (3.07%)	1098 (2.83%)	-1.42	235 (1.43%)	222 (1.35%)	-0.68
	Hemiplegia*	3441 (7.63%)	4026 (8.92%)	4.71	2700 (6.97%)	2680 (6.92%)	-0.2	1460 (8.89%)	1436 (8.75%)	-0.52
	Moderate or severe renal disease	10422 (23.1%)	9050 (20.06%)	-7.4	5497 (14.19%)	4456 (11.5%)	-8.04	870 (5.3%)	729 (4.44%)	-3.99
	Any tumor (except for malignant neoplasm of skin)*	4590 (10.17%)	5116 (11.34%)	3.76	3775 (9.75%)	4175 (10.78%)	3.4	1379 (8.4%)	1357 (8.27%)	-0.48
	Metastatic solid tumor*	930 (2.06%)	1063 (2.36%)	2.01	824 (2.13%)	917 (2.37%)	1.62	300 (1.83%)	281 (1.71%)	-0.88
	HIV/AIDS*	72 (0.16%)	70 (0.16%)	-0.11	62 (0.16%)	66 (0.17%)	0.25	10 (0.06%)	12 (0.07%)	0.47
	Moderate or severe liver disease*	325 (0.72%)	295 (0.65%)	-0.8	247 (0.64%)	215 (0.56%)	-1.07	56 (0.34%)	55 (0.34%)	-0.1
**CHA**_**2**_ **DS**_**2**_ **-VASc score**	Mean (SD)	4.4 [1.4]	4.4 [1.5]		4.2 [1.4]	4.2 [1.5]		3.8 [1.4]	3.8 [1.5]	
	0	193 (0.43%)	309 (0.68%)	3.46	191 (0.49%)	327 (0.84%)	4.31	174 (1.06%)	251 (1.53%)	4.15
	1	839 (1.86%)	1106 (2.45%)	4.08	829 (2.14%)	1224 (3.16%)	6.35	641 (3.9%)	760 (4.63%)	3.59
	2–3	10436 (23.1%)	10601 (23.5%)		10007 (25.8%)	10387 (26.8%)		6115 (37.25%)	5959 (36.3%)	
	≥4	33656 (74.59%)	33108 (73.37%)	-2.77	27710 (71.53%)	26799 (69.18%)	-5.15	9485 (57.78%)	9445 (57.54%)	-0.49
**Concomitant treatment**	Antiplatelets	22216 (49.23%)	23132 (51.26%)	4.06	19142 (49.42%)	19628 (50.67%)	2.51	7830 (47.7%)	7810 (47.58%)	-0.32
	Aromatase inhibitors*	336 (0.74%)	385 (0.85%)	1.22	286 (0.74%)	324 (0.84%)	1.11	107 (0.65%)	105 (0.64%)	-0.15
	NSAIDs	2648 (5.87%)	3581 (7.94%)	8.16	2457 (6.34%)	3344 (8.63%)	8.71	1595 (9.72%)	1843 (11.23%)	4.85
	H2-receptor antagonists*	212 (0.47%)	268 (0.59%)	1.71	185 (0.48%)	210 (0.54%)	0.91	65 (0.4%)	70 (0.43%)	0.48
	Prostaglandins	624 (1.38%)	829 (1.84%)	3.61	510 (1.32%)	918 (2.37%)	7.84	251 (1.53%)	355 (2.16%)	4.71
	Proton pump inhibitors*	24287 (53.82%)	24876 (55.13%)	2.62	20188 (52.12%)	20742 (53.55%)	2.87	7611 (46.37%)	7409 (45.14%)	-2.47
	Anticonvulsant strong inhibitor of hepatic enzymes*	359 (0.8%)	402 (0.89%)	1.04	313 (0.81%)	338 (0.87%)	0.71	945 (0.57%)	93 (0.57%)	-0.08
	HIV protease inhibitors*	139 (0.31%)	182 (0.4%)	1.6	111 (0.29%)	155 (0.4%)	1.94	60 (0.37%)	55 (0.34%)	-0.52
	Strong inhibitors of both CYP3A4 and P-gp*	532 (1.18%)	646 (1.43%)	2.23	450 (1.16%)	591 (1.53%)	3.16	245 (1.49%)	255 (1.55%)	0.5
	Statins*	7337 (16.26%)	8090 (17.93%)	4.43	6393 (16.5%)	7091 (18.31%)	4.75	2810 (17.12%)	2764 (16.84%)	-0.75
	Selective estrogen receptor modulators*	79 (0.18%)	97 (0.21%)	0.9	69 (0.18%)	76 (0.2%)	0.42	30 (0.18%)	32 (0.19%)	0.28
	Serotonin reuptake inhibitors*	4220 (9.35%)	4845 (10.74%)	4.61	3549 (9.16%)	3886 (10.03%)	2.95	1400 (8.53%)	1306 (7.96%)	-2.08
	Sex hormones	924 (2.05%)	1263 (2.8%)	4.89	784 (2.02%)	1287 (3.32%)	8.06	406 (2.47%)	544 (3.31%)	5.02
	Erythropoesis stimulating agents*	714 (1.58%)	613 (1.36%)	-1.86	351 (0.91%)	305 (0.79%)	-1.3	45 (0.27%)	46 (0.28%)	0.12
	Beta blockers*	27802 (61.61%)	27360 (60.63%)	-2.01	23637 (61.02%)	23365 (60.32%)	-1.44	9176 (55.9%)	9209 (56.1%)	0.4
	Antiarrhythmic agents*	21627 (47.93%)	21434 (47.5%)	-0.86	19302 (49.83%)	19188 (49.53%)	-0.59	9865 (60.1%)	9889 (60.24%)	0.3

*Variables that were adjusted for in the PS model

^†^Censored at switch, discontinuation, interruption, death, pregnancy, dialysis, chronic kidney disease stage V or end of follow-up

^‡^The following age categories were used to match API–VKA: 18–54 / 55–64 / 65–74 / 75–79 / 80–84 / 85–89 / ≥90 years and a more refined one was considered for the other treatment groups to obtain a good match (standard difference*100 lower than 10) on age (18–54 / 55–64 / 65–74 / 75–79 / 80–84 / 85–89 / 90–94 / ≥95 years). Scores (Charlson Comorbidity Index, HAS-BLED and CHA_2_DS_2_-VASc) were not included in the PS modelling as their components are singularly included, but were used as indicator for evaluating the fitness of the matching. AIDS, acquired immunodeficiency syndrome; CYP3A4, cytochrome P450 3A4; DOAC, direct oral anticoagulant; GIB, gastrointestinal bleed; HIV, human immunodeficiency virus; IQR, interquartile range; LTR, long-term recurrence; NSAID, nonsteroidal anti-inflammatory drug; P-gp, P-glycoprotein; PS, propensity score; SD, standard deviation; VKA, vitamin K antagonist.

**Table 2:** Baseline characteristics of patients prescribed DOACs versus DOACs after PS matching.

Characteristic		Apixaban versus rivaroxaban			Apixaban versus dabigatran			Rivaroxaban versus dabigatran		
		Apixaban (n = 88,414)	Rivaroxaban (n = 88,414)	Standardized difference	Apixaban (n = 16,464)	Dabigatran (n = 16,464)	Standardized difference	Rivaroxaban (n = 16,459)	Dabigatran (n = 16,459)	Standardized difference
**Atrial fibrillation identification setting**	Inpatient claim with I48 code	49098 (55.53%)	49620 (56.12%)	1.19	8986 (54.58%)	9253 (56.2%)	3.26	8932 (54.27%)	9247 (56.18%)	3.85
	LTR registration with I48 code*	10172 (11.5%)	9960 (11.27%)	-0.75	1983 (12.04%)	1904 (11.56%)	-1.49	2002 (12.16%)	1904 (11.57%)	-1.84
	Use of anti-arrhythmic drugs*	29144 (32.96%)	28834 (32.61%)	-0.75	5495 (33.38%)	5307 (32.23%)	-2.43	5525 (33.57%)	5308 (32.25%)	-2.81
**Follow up time (months)** ^†^	Mean (SD)	13.8 (12.8)	13.0 (12.7)		14.0 (13.0)	13.5 (12.7)		13.2 (12.9)	13.5 (12.7)	
	Median (IQR)	9.2 (3.1–21.8)	8.2 (2.6–21.0)		9.5 (3.1–22.4)	9.0 (2.5–22.7)		8.3 (2.5–21.3)	9.0 (2.5–22.7)	
**Index dosage**	Standard	56700 (64.13%)	56125 (63.48%)	-1.35	5766 (35.02%)	5699 (34.61%)	-0.85	5736 (34.85%)	5699 (34.63%)	-0.47
	Reduced*	31714 (35.87%)	32289 (36.52%)		10698 (64.98%)	10765 (65.39%)		10723 (65.15%)	10760 (65.37%)	
**Age at index date (years)**		76.9 [10.6]	76.6 [10.8]	-2.75	78 [10.1]	77.8 [10.1]	-1.55	77.8 [10.1]	77.8 [10.1]	0.02
**Age groups at index date**	18–54 years	3044 (3.44%)	3348 (3.79%)	1.84	401 (2.44%)	392 (2.38%)	-0.36	366 (2.22%)	392 (2.38%)	1.05
	55–64 years*	8023 (9.07%)	8272 (9.36%)	0.97	1271 (7.72%)	1267 (7.7%)	-0.09	1278 (7.76%)	1267 (7.7%)	-0.25
	65–74 years*	19735 (22.32%)	19775 (22.37%)	0.11	3543 (21.52%)	3519 (21.37%)	-0.36	3611 (21.94%)	3516 (21.36%)	-1.4
	75–79 years*	17661 (19.98%)	17456 (19.74%)	-0.58	3147 (19.11%)	3182 (19.33%)	0.54	3172 (19.27%)	3180 (19.32%)	0.12
	80–84 years*	18265 (20.66%)	18012 (20.37%)	-0.71	3560 (21.62%)	3631 (22.05%)	1.04	3621 (22.00%)	3631 (22.06%)	0.15
	85–89 years*	14036 (15.88%)	13983 (15.82%)	-0.16	2997 (18.20%)	2935 (17.83%)	-0.98	2885 (17.53%)	2935 (17.83%)	0.80
	90–94 years*	6109 (6.91%)	6116 (6.92%)	0.03	1277 (7.76%)	1258 (7.64%)	-0.43	1262 (7.67%)	1258 (7.64%)	-0.09
	≥95 years*	1541 (1.74%)	1452 (1.64%)	-0.78	268 (1.63%)	280 (1.70%)	0.57	264 (1.60%)	280 (1.70%)	0.76
									
**Sex**	Male	47037 (53.2%)	47176 (53.4%)		8361 (50.8%)	8478 (51.5%)		8448 (51.3%)	8475 (51.5%)	
	Female*	41377 (46.8%)	41238 (46.64%)	0.32	8103 (49.22%)	7986 (48.51%)	1.42	8011 (48.67%)	7984 (48.51%)	0.33
**GIB risk factors**	Age ≥75 years	57612 (65.16%)	57019 (64.49%)	-1.4	11249 (68.32%)	11286 (68.55%)	0.48	11204 (68.07%)	11284 (68.56%)	1.04
	HAS-BLED score, mean	2.5 [1]	2.5 [1]		2.6 [1]	2.6 [1]		2.6 [1]	2.6 [1]	
	0	888 (1%)	1011 (1.14%)	1.35	134 (0.81%)	114 (0.69%)	-1.41	136 (0.83%)	114 (0.69%)	-1.54
	1	10469 (11.84%)	10801 (12.22%)	1.15	1826 (11.09%)	1754 (10.65%)	-1.4	1803 (10.95%)	1754 (10.66%)	-0.96
	2	30256 (34.22%)	29756 (33.66%)	-1.19	5326 (32.35%)	5263 (31.97%)	-0.82	5294 (32.16%)	5262 (31.97%)	-0.42
	≥3*	46801 (52.93%)	46846 (52.98%)	0.1	9178 (55.75%)	9333 (56.69%)	1.9	9226 (56.05%)	9329 (56.68%)	1.26
	Concomitant medications (NSAIDs, antiplatelets, corticosteroids)	57002 (64.47%)	57568 (65.11%)	1.34	10157 (61.69%)	10145 (61.62%)	-0.15	10222 (62.11%)	10142 (61.62%)	-1.0
	Renal impairment	2636 (2.98%)	2655 (3%)	0.13	377 (2.29%)	395 (2.4%)	0.72	363 (2.21%)	395 (2.4%)	1.3
	Prior GI condition	6838 (7.73%)	7059 (7.98%)	0.93	1238 (7.52%)	1374 (8.35%)	3.06	1265 (7.69%)	1371 (8.33%)	2.37
**Number of GIB risk factors**	1	37068 (41.93%)	36941 (41.78%)	-0.29	6595 (40.06%)	6507 (39.52%)	-1.09	6576 (39.95%)	6507 (39.53%)	-0.86
	2	23129 (26.16%)	23130 (26.16%)	0	4498 (27.32%)	4426 (26.88%)	-0.98	4421 (26.86%)	4423 (26.87%)	0.03
	3	25424 (28.76%)	25555 (28.9%)	0.33	4890 (29.7%)	4975 (30.22%)	1.13	5001 (30.38%)	4973 (30.21%)	-0.37
	4	2674 (3.02%)	2659 (3.01%)	-0.1	467 (2.84%)	531 (3.23%)	2.27	446 (2.71%)	531 (3.23%)	3.04
	5	119 (0.13%)	129 (0.15%)	0.3	14 (0.09%)	25 (0.15%)	1.94	15 (0.09%)	25 (0.15%)	1.74
**Charlson Comorbidity Index score**	0	32623 (36.9%)	32542 (36.81%)	-0.19	6139 (37.29%)	5777 (35.09%)	-4.58	6060 (36.82%)	5777 (35.1%)	-3.58
	1 or 2	38933 (44.03%)	38616 (43.68%)	-0.72	6858 (41.65%)	6971 (42.34%)	1.39	6860 (41.68%)	6970 (42.35%)	1.35
	3 or 4	11504 (13.01%)	11788 (13.33%)	0.95	2453 (14.9%)	2559 (15.54%)	1.79	2452 (14.9%)	2558 (15.54%)	1.79
	≥5	5354 (6.06%)	5468 (6.18%)	0.54	1014 (6.16%)	1157 (7.03%)	3.5	1087 (6.6%)	1154 (7.01%)	1.62
**Additional comorbidities**	Myocardial infarction*	6050 (6.84%)	6144 (6.95%)	0.42	863 (5.24%)	886 (5.38%)	0.62	885 (5.38%)	886 (5.38%)	0.03
	Congestive heart failure*	21477 (24.29%)	21767 (24.62%)	0.76	3404 (20.68%)	3622 (22.0%)	3.23	3549 (21.56%)	3621 (22.0%)	1.06
	Peripheral vascular disease*	5968 (6.75%)	6238 (7.06%)	1.2	1001 (6.08%)	1110 (6.74%)	2.7	1028 (6.25%)	1109 (6.74%)	2.0
	Cerebrovascular disease*	9794 (11.08%)	9800 (11.08%)	0.02	2938 (17.84%)	3145 (19.1%)	3.24	3099 (18.83%)	3141 (19.08%)	0.65
	Dementia*	4966 (5.62%)	5115 (5.79%)	0.73	811 (4.93%)	847 (5.14%)	1	801 (4.87%)	846 (5.14%)	1.25
	Chronic pulmonary disease*	17760 (20.09%)	17914 (20.26%)	0.43	2981 (18.11%)	3133 (19.03%)	2.37	801 (4.87%)	846 (5.14%)	2.31
	Connective tissue disease*	1099 (1.24%)	1128 (1.28%)	0.29	195 (1.18%)	206 (1.25%)	0.61	201 (1.22%)	206 (1.25%)	0.27
	Ulcer disease*	647 (0.73%)	673 (0.76%)	0.34	136 (0.83%)	159 (0.97%)	1.48	151 (0.92%)	158 (0.96%)	0.44
	Mild liver disease*	1178 (1.33%)	1236 (1.4%)	0.57	164 (1%)	218 (1.32%)	3.06	182 (1.11%)	217 (1.32%)	1.94
	Diabetes*	17331 (19.6%)	17383 (19.66%)	0.15	3005 (18.25%)	3074 (18.67%)	1.08	3028 (18.4%)	3073 (18.67%)	0.7
	Diabetes with end-organ damage*	1243 (1.41%)	1221 (1.38%)	-0.21	209 (1.27%)	222 (1.35%)	0.69	191 (1.16%)	222 (1.35%)	1.69
	Hemiplegia*	3603 (4.08%)	3469 (3.92%)	-0.77	1295 (7.87%)	1437 (8.73%)	3.13	1415 (8.6%)	1433 (8.71%)	0.39
	Moderate or severe renal disease	4672 (5.28%)	4674 (5.29%)	0.01	698 (4.24%)	729 (4.43%)	0.92	687 (4.17%)	729 (4.43%)	1.26
	Any tumor (except for malignant neoplasm of skin)*	6463 (7.31%)	6729 (7.61%)	1.15	1238 (7.52%)	1357 (8.24%)	2.68	1263 (7.67%)	1356 (8.24%)	2.09
	Metastatic solid tumor*	1346 (1.52%)	1400 (1.58%)	0.49	261 (1.59%)	281 (1.71%)	0.95	253 (1.54%)	281 (1.71%)	1.35
	HIV/ AIDS*	64 (0.07%)	73 (0.08%)	0.37	13 (0.08%)	12 (0.07%)	-0.22	8 (0.05%)	12 (0.07%)	0.99
	Moderate or severe liver disease*	234 (0.26%)	237 (0.27%)	0.07	38 (0.23%)	55 (0.33%)	1.95	55 (0.33%)	54 (0.33%)	-0.11
**CHA**_**2**_ **DS**_**2**_ **-VASc score**	Mean (SD)	3.6 [1.5]	3.6 [1.5]		3.7 [1.5]	3.8 [1.5]		3.7 [1.5]	3.8 [1.5]	
	0	1753 (1.98%)	2211 (2.5%)	3.5	302 (1.83%)	256 (1.55%)	-2.16	298 (1.81%)	256 (1.56%)	-1.98
	1	5139 (5.81%)	5499 (6.22%)	1.71	783 (4.76%)	772 (4.69%)	-0.31	828 (5.03%)	772 (4.69%)	-1.58
	2–3	34957 (39.5%)	34411 (38.9%)		6186 (37.6%)	5988 (36.4%)		6153 (37.4%)	5987 (36.4%)	
	≥4	46565 (52.67%)	46293 (52.36%)	-0.62	9193 (55.84%)	9448 (57.39%)	3.13	9180 (55.77%)	9444 (57.38%)	3.24
**Concomitant treatment**	Antiplatelets	43667 (49.39%)	43614 (49.33%)	-0.12	7832 (47.57%)	7830 (47.56%)	-0.02	7980 (48.48%)	7829 (47.57%)	-1.84
	Aromatase inhibitors*	610 (0.69%)	605 (0.68%)	-0.07	103 (0.63%)	105 (0.64%)	0.15	127 (0.77%)	105 (0.64%)	-1.6
	NSAIDs	10905 (12.33%)	11124 (12.58%)	0.75	1908 (11.59%)	1876 (11.39%)	-0.61	1814 (11.02%)	1876 (11.4%)	1.19
	H2-receptor antagonists*	349 (0.39%)	358 (0.4%)	0.16	69 (0.42%)	70 (0.43%)	0.09	53 (0.32%)	70 (0.43%)	1.69
	Prostaglandins	2534 (2.87%)	2321 (2.63%)	-1.47	425 (2.58%)	357 (2.17%)	-2.71	325 (1.97%)	357 (2.17%)	1.36
	Proton pump inhibitors*	38460 (43.5%)	38515 (43.56%)	0.13	7352 (44.66%)	7432 (45.14%)	0.98	7357 (44.7%)	7428 (45.13%)	0.87
	Anticonvulsant strong inhibitor of hepatic enzymes*	494 (0.56%)	521 (0.59%)	0.4	96 (0.58%)	93 (0.56%)	-0.24	78 (0.47%)	93 (0.57%)	1.27
	HIV protease inhibitors*	326 (0.37%)	349 (0.39%)	0.42	56 (0.34%)	55 (0.33%)	-0.1	47 (0.29%)	55 (0.33%)	0.87
	Strong inhibitors of both CYP3A4 and P-gp*	1370 (1.55%)	1387 (1.57%)	0.16	233 (1.42%)	258 (1.57%)	1.25	203 (1.23%)	257 (1.56%)	2.8
	Statins*	14555 (16.46%)	14651 (16.57%)	0.29	2688 (16.33%)	2772 (16.84%)	1.37	2673 (16.24%)	2771 (16.84%)	1.6
	Selective estrogen receptor modulators*	164 (0.19%)	166 (0.19%)	0.05	39 (0.24%)	32 (0.19%)	-0.92	28 (0.17%)	32 (0.19%)	0.57
	Serotonin reuptake inhibitors*	6168 (6.98%)	6402 (7.24%)	1.03	1226 (7.45%)	1307 (7.94%)	1.85	1230 (7.47%)	1307 (7.94%)	1.75
	Sex hormones	3547 (4.01%)	3420 (3.87%)	-0.74	617 (3.75%)	546 (3.32%)	-2.34	527 (3.2%)	546 (3.32%)	0.65
	Erythropoesis stimulating agents*	281 (0.32%)	322 (0.36%)	0.8	47 (0.29%)	46 (0.28%)	-0.11	56 (0.34%)	46 (0.28%)	-1.09
	Beta blockers*	50665 (57.3%)	50529 (57.15%)	-0.31	9165 (55.67%)	9225 (56.03%)	0.73	9190 (55.84%)	9221 (56.02%)	0.38
	Antiarrhythmic agents*	54221 (61.33%)	54125 (61.22%)	-0.22	10088 (61.27%)	9930 (60.31%)	-1.97	10256 (62.31%)	9928 (60.32%)	-4.09

*Variables that were adjusted for in the PS model

^†^Censored at switch, discontinuation, interruption, death, pregnancy, dialysis, chronic kidney disease stage V or end of follow-up. Scores (Charlson Comorbidity Index, HAS-BLED and CHA_2_DS_2_-VASc) were not included in the PS modelling as their components are singularly included, but were used as indicator for evaluating the fitness of the matching. AIDS, acquired immunodeficiency syndrome; CYP3A4, cytochrome P450 3A4; DOAC, direct oral anticoagulant; GIB, gastrointestinal bleed; HIV, human immunodeficiency virus; IQR, interquartile range; LTR, long-term recurrence; NSAID, nonsteroidal anti-inflammatory drug; P-gp, P-glycoprotein; PS, propensity score; SD, standard deviation; VKA, vitamin K antagonist.

#### Time-to-event analyses

**Fig 2:**
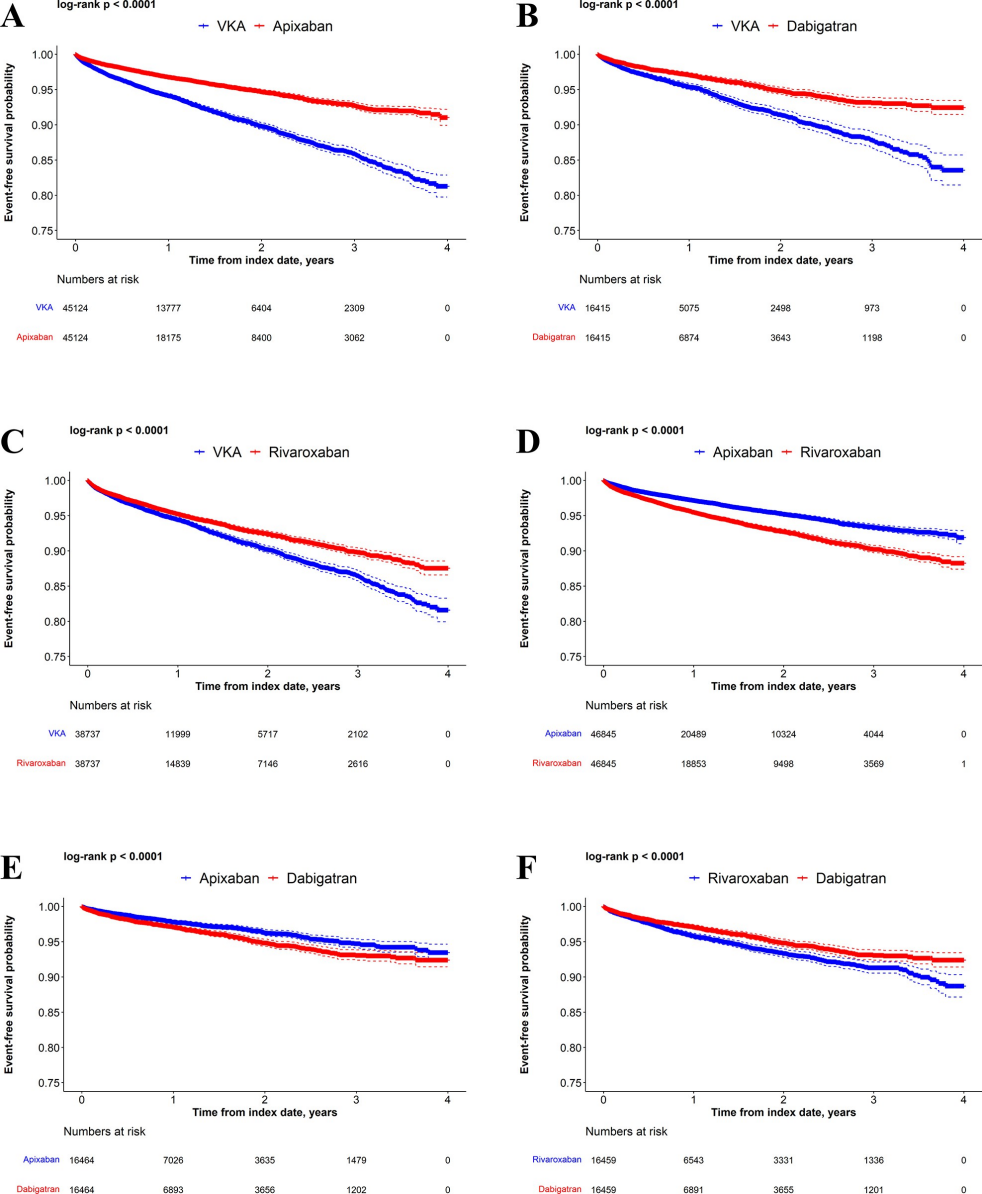
Kaplan-Meier curves for MB. Apixaban versus VKAs (A), dabigatran versus VKAs (B), rivaroxaban versus VKAs (C), rivaroxaban versus apixaban (D), dabigatran versus apixaban (E), and dabigatran versus rivaroxaban (F).

**Fig 3:**
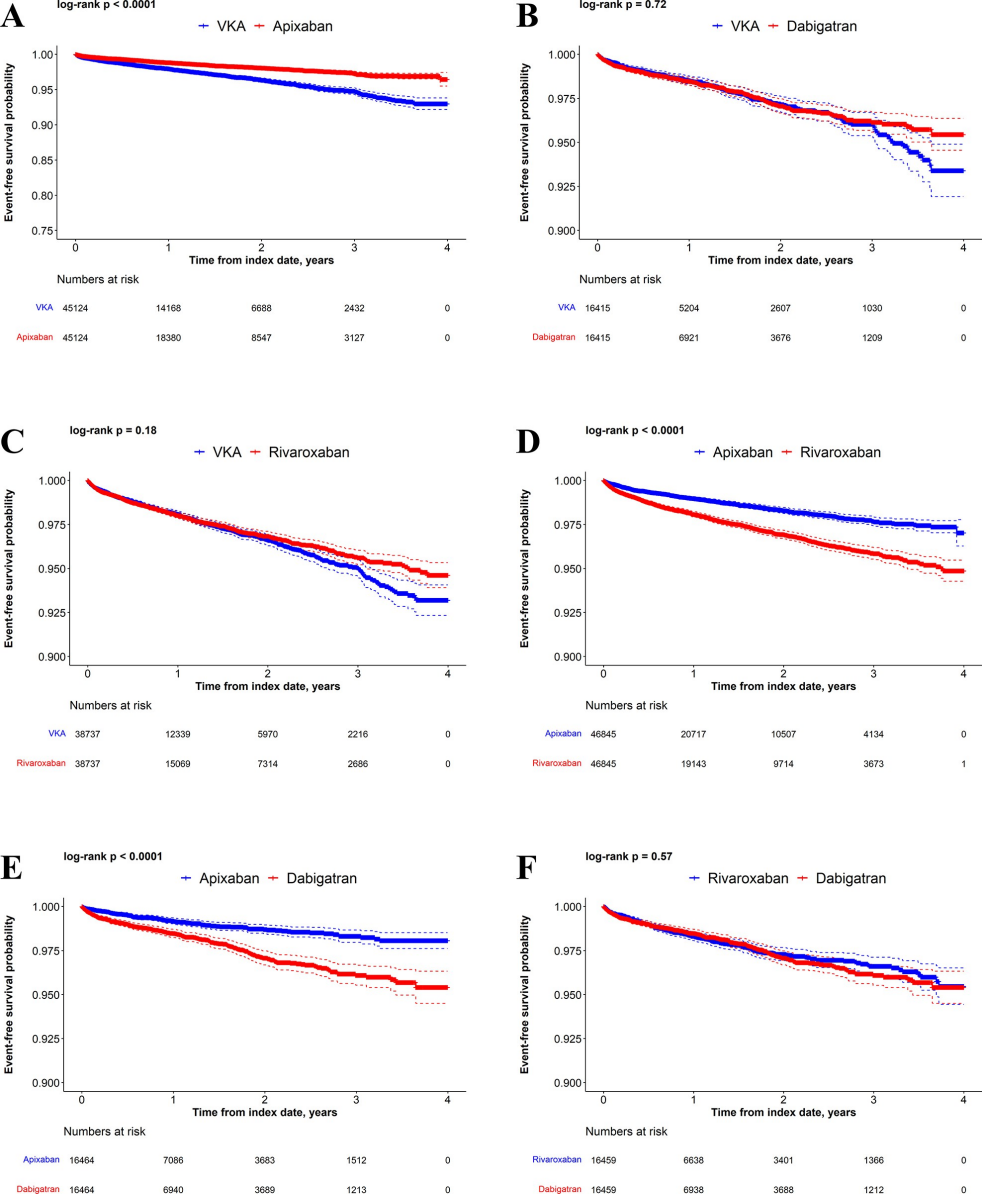
Kaplan-Meier curves for GIB. Apixaban versus VKAs (A), dabigatran versus VKAs (B), rivaroxaban versus VKAs (C), rivaroxaban versus apixaban (D), dabigatran versus apixaban (E), and dabigatran versus rivaroxaban (F).

**Fig 4:**
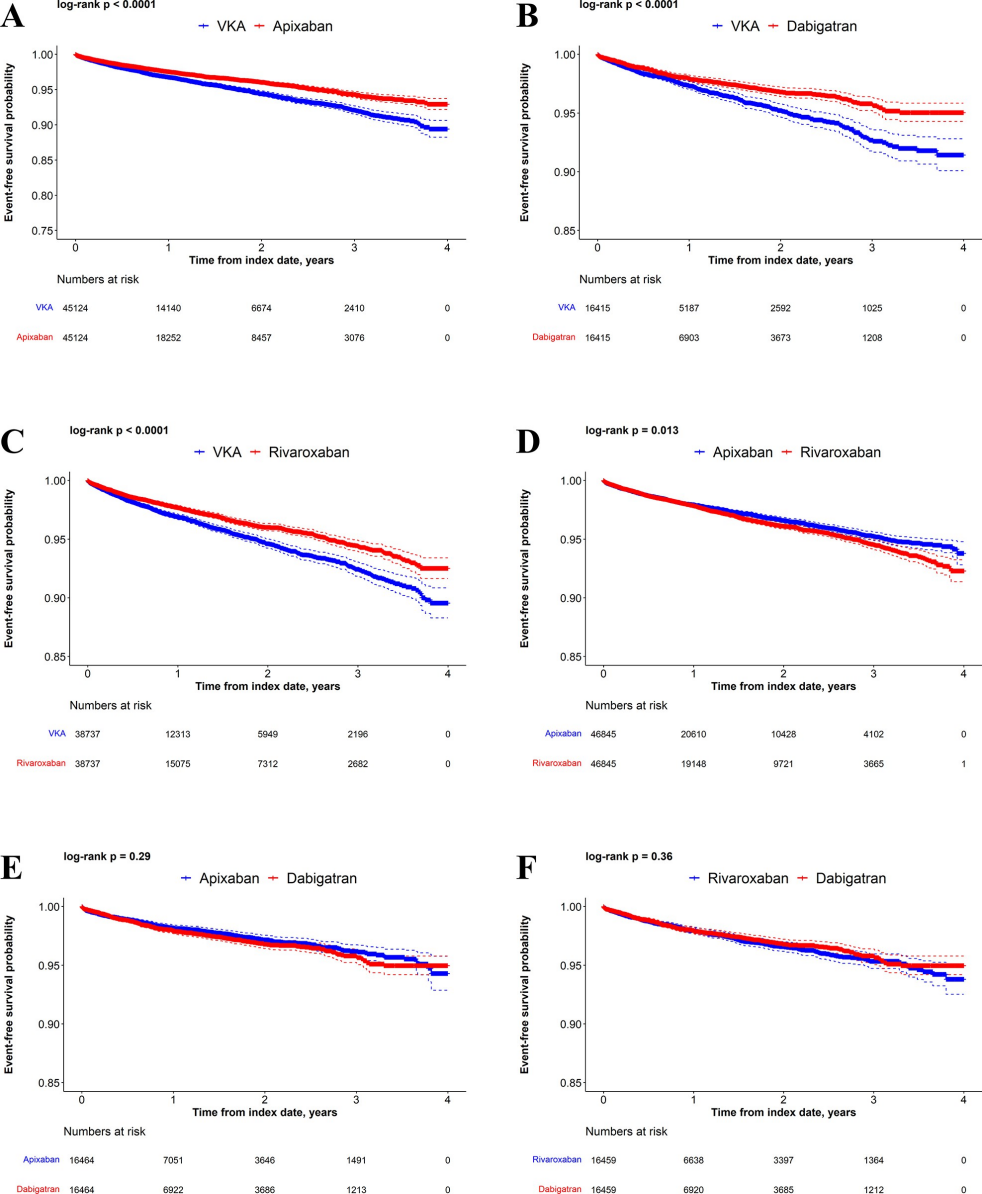
Kaplan-Meier curves for stroke/SE. Apixaban versus VKAs (A), dabigatran versus VKAs (B), rivaroxaban versus VKAs (C), rivaroxaban versus apixaban (D), dabigatran versus apixaban (E), and dabigatran versus rivaroxaban (F).

Kaplan-Meier curves comparing VKAs versus DOACs and DOACs versus DOACs in the matched populations are presented for time to MB (**Fig 2**), time to GIB (**Fig 3**), and time to stroke/SE (**Fig 4**). Kaplan-Meier curves for cumulative incidence of stroke and/or SE and other MB in the matched populations appear in **S1–S6 Figs**.

#### VKA versus DOAC

**Fig 5:**
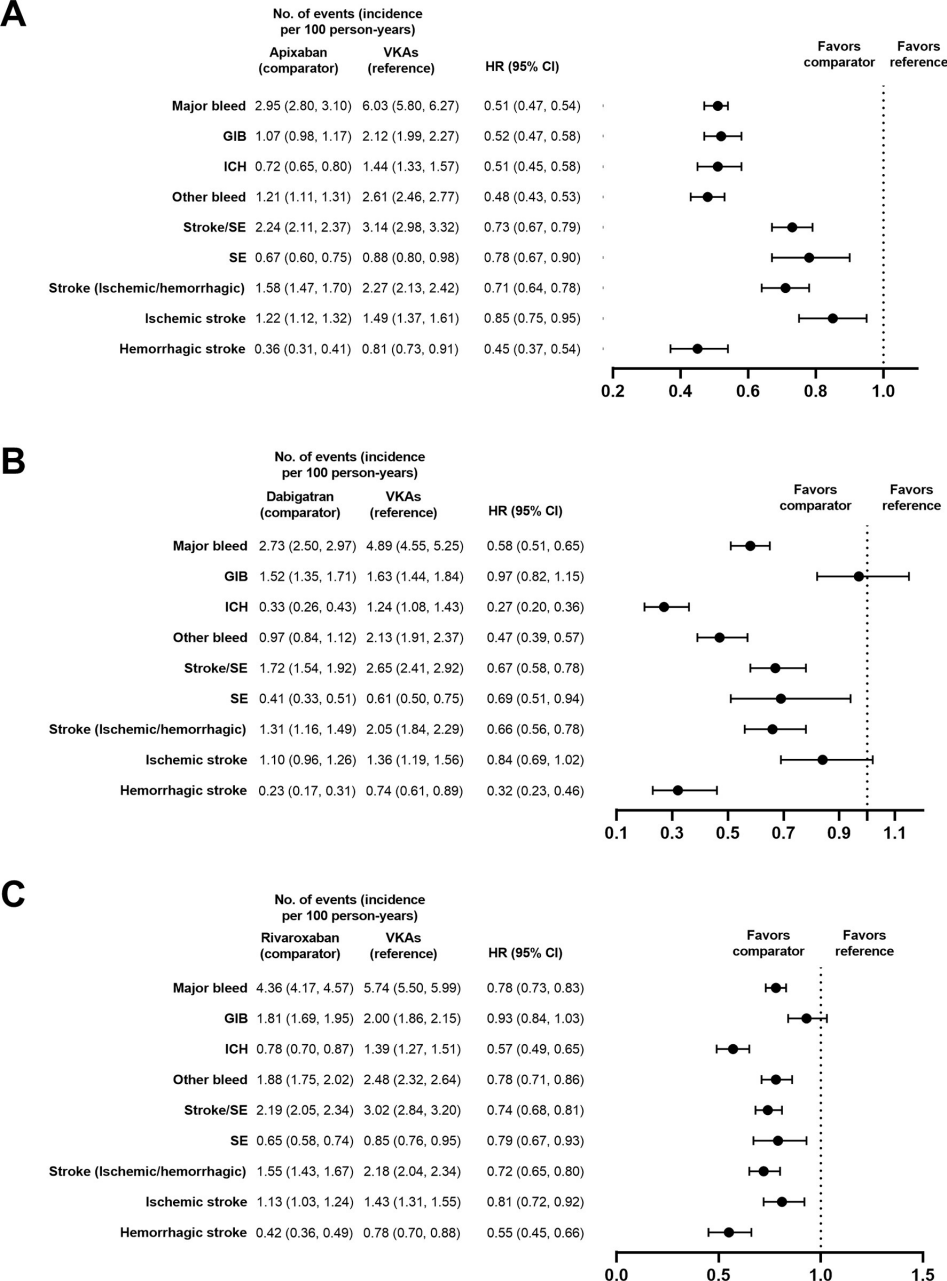
PS-matched hazard ratios for DOACs versus VKAs. Apixaban (A), dabigatran (B), and rivaroxaban (C).

After PS matching, apixaban (HR, 0.51, 95% CI, 0.47–0.54), rivaroxaban (HR, 0.78; 95% CI, 0.73–0.83), and dabigatran (HR, 0.58; 95% CI, 0.51–0.65) use were associated with a lower risk of MB leading to hospitalization than VKAs (*p*<0.0001 for all; **Fig 5**). Patients receiving apixaban had a lower risk of GIB versus those receiving VKAs (HR, 0.52; 95% CI, 0.47–0.58; *p*<0.0001; **Fig 5**). Neither rivaroxaban (HR, 0.93; 95% CI, 0.84–1.03; *p* = 0.18) nor dabigatran (HR, 0.97; 95% CI, 0.82–1.15; *p* = 0.72) were associated with risk of GIB versus VKAs. DOACs similarly were associated with a lower risk of ICH and other bleeds than VKAs (**Fig 5**).

Apixaban (HR, 0.73, 95% CI, 0.67–0.79), rivaroxaban (HR, 0.74; 95% CI, 0.68–0.81), and dabigatran (HR, 0.67; 95% CI, 0.58–0.78) use were associated with lower risk of stroke/SE versus VKAs (*p*<0.0001 for all).

#### DOAC comparisons

**Fig 6:**
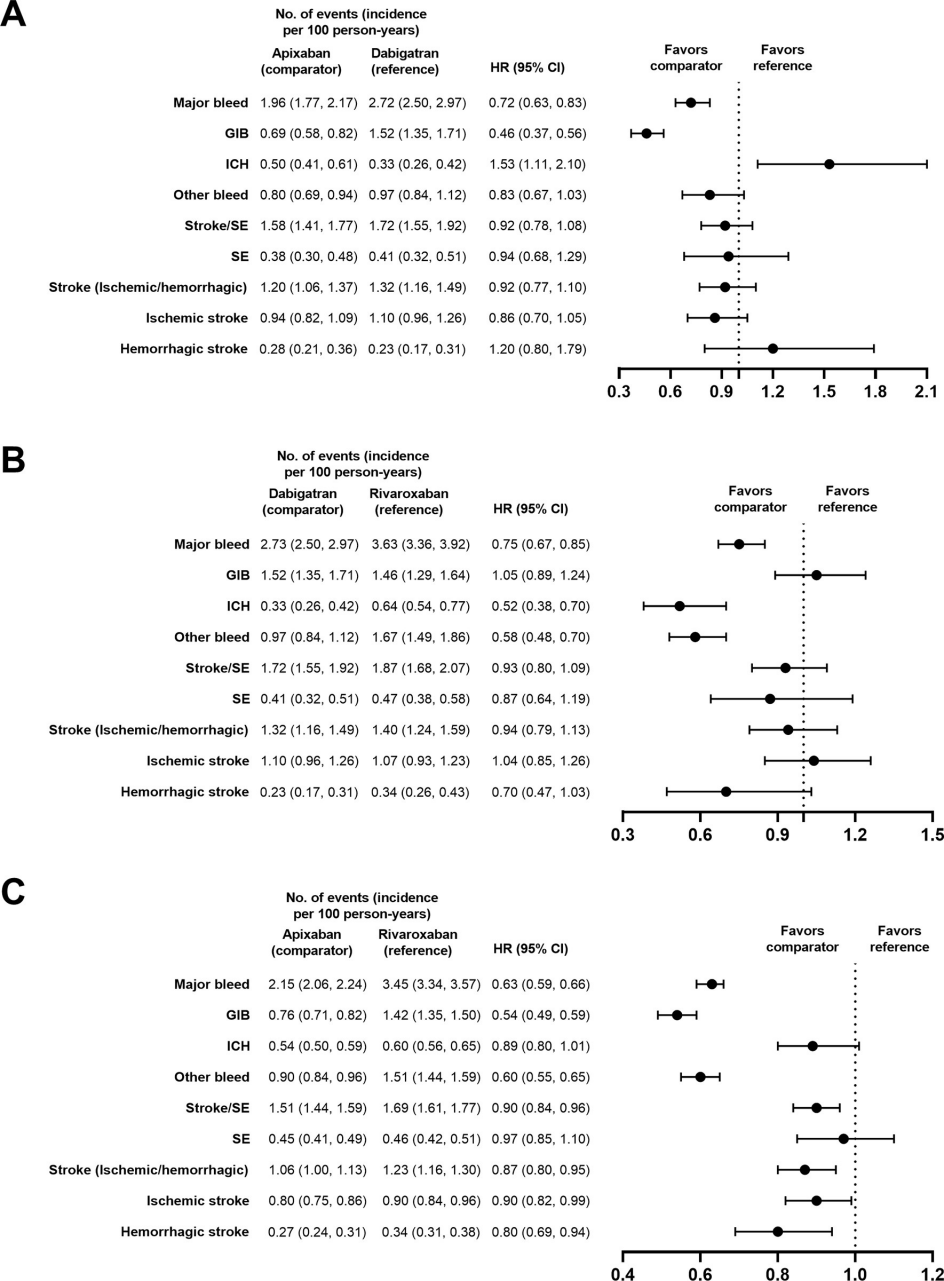
PS-matched hazard ratios for DOACs versus DOACs. Apixaban versus dabigatran (A), dabigatran versus rivaroxaban (B), and apixaban versus rivaroxaban (C).

Apixaban use was associated with a lower risk of MB versus dabigatran (HR, 0.72; 95% CI, 0.63–0.83) and rivaroxaban (HR, 0.63; 95% CI, 0.59–0.66; **Fig 6**). Apixaban use was also associated with a lower risk of GIB versus dabigatran (HR, 0.46; 95% CI, 0.37–0.56) and rivaroxaban (HR, 0.54; 95% CI, 0.49–0.59; **Fig 6**). Dabigatran use was associated with a similar risk of GIB versus rivaroxaban (HR, 1.05; 95% CI, 0.89–1.24). Compared with rivaroxaban, dabigatran use was associated with a lower risk of ICH (HR, 0.52; 95% CI, 0.38–0.70; **Fig 6**). Apixaban use was also associated with a higher risk of ICH versus dabigatran (HR, 1.53; 95% CI, 1.11–2.10).

Compared with rivaroxaban, apixaban use was associated with a lower risk of stroke/SE (HR, 0.90; 95% CI, 0.84–0.96; **Fig 6**). Risk of stroke/SE was similar when comparing apixaban with dabigatran (HR, 0.92; 95% CI, 0.78–1.08) and dabigatran with rivaroxaban (HR, 0.93; 95% CI, 0.80–1.09).

#### Subgroup analysis

**Fig 7:**
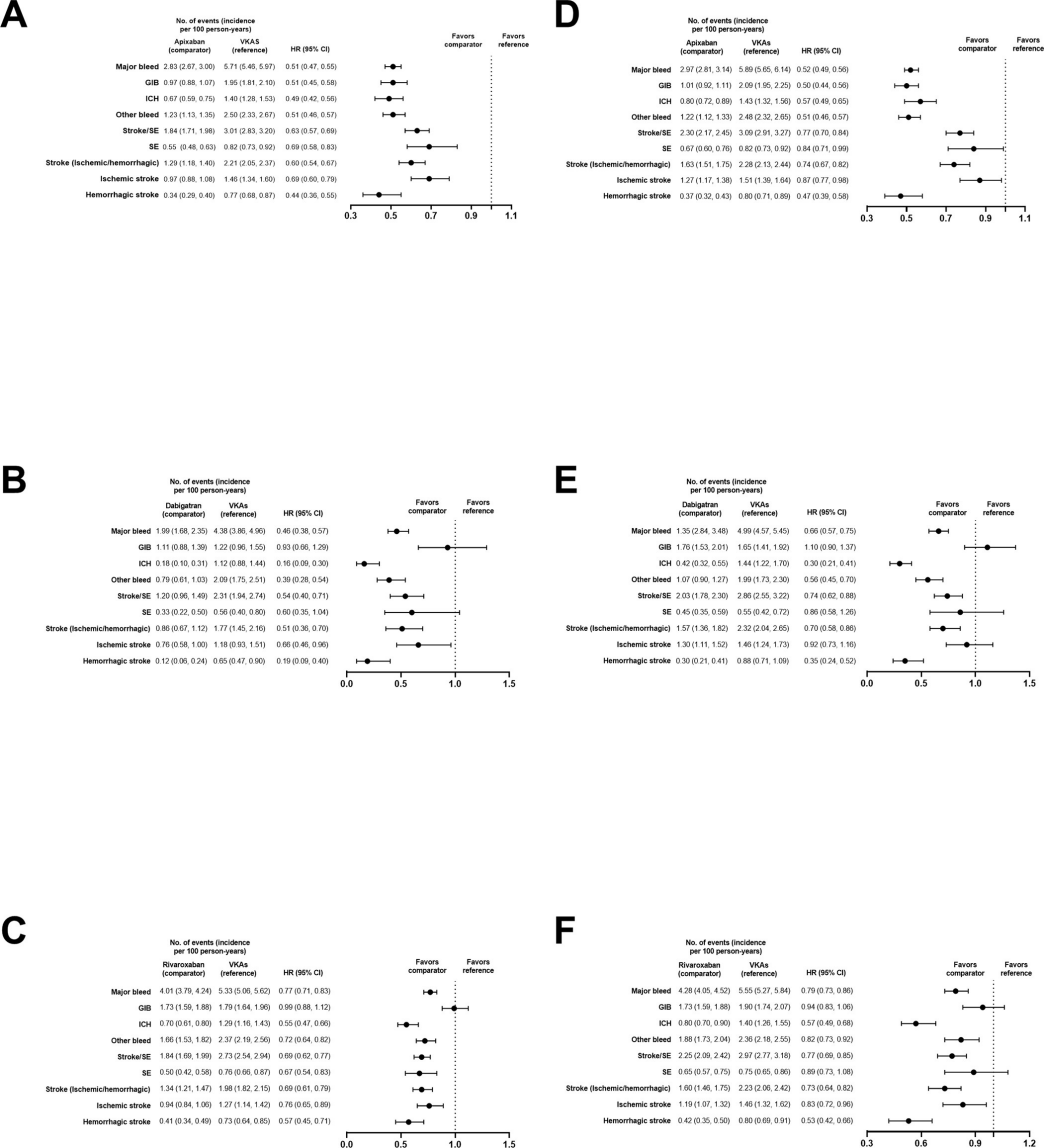
PS-matched hazard ratios for subgroup analyses of DOACs versus VKAs. Standard dose of apixaban (A), dabigatran (B), and rivaroxaban (C) and reduced dose of apixaban (D), dabigatran (E), and rivaroxaban (F).

**Fig 8:**
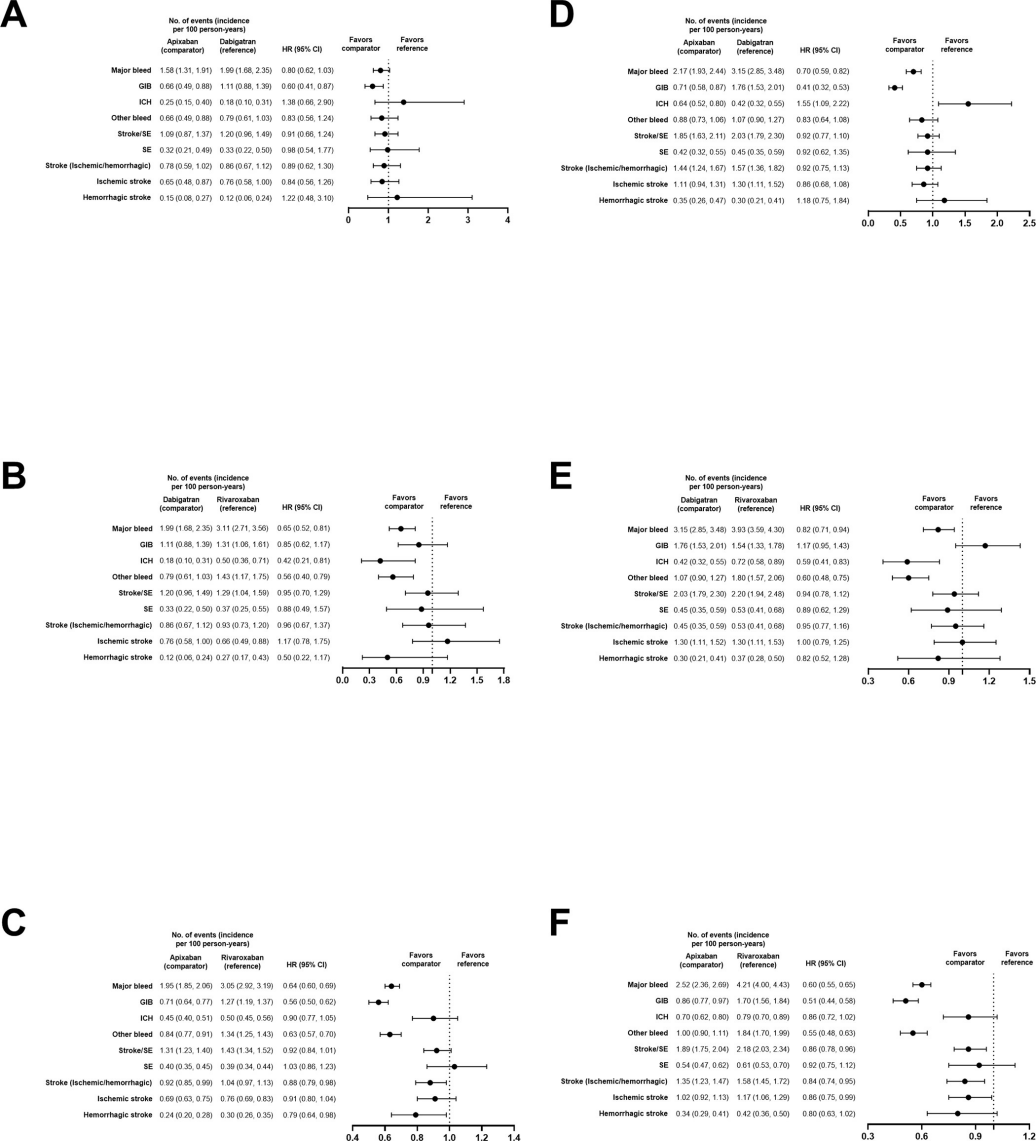
PS-matched hazard ratios for subgroup analyses of DOACs versus DOACs. Standard dose of apixaban versus dabigatran (A), dabigatran versus rivaroxaban (B), and apixaban versus rivaroxaban (C) and reduced dose of apixaban versus dabigatran (D), dabigatran versus rivaroxaban (E), and apixaban versus rivaroxaban (F).

Results of analyses evaluating the standard and reduced doses were generally consistent with the main analysis for MB and all other outcomes of interest in both DOAC–VKA and DOAC–DOAC comparisons (**Figs 7 and 8**).

#### Sensitivity analyses

Results were also similar after excluding patients with probable AF (**S7 Fig**). Risk of ICH was similar between apixaban and dabigatran (HR, 1.38; 95% CI, 0.66–2.9). Risk of other bleeding was lower with apixaban versus rivaroxaban (HR 0.60; 95% CI, 0.55–0.66) whereas risk of ischemic stroke was similar between the two (HR 0.91; 95% CI, 0.8–1.04).

Results of AFT analyses were similar to the main analyses using Cox proportional hazards models. Estimated relative acceleration factors favored DOACs versus VKAs for risk of MB (*p*<0.0001 for all) as well as most outcomes of interest (**S6 Table**).

## Discussion

Data evaluating OACs are limited in the population of patients with NVAF at high risk for GIB, as studies tend to assess individual risk factors. Here we evaluated the effectiveness and safety of DOACs using a combination of validated and published risk factors to provide a comprehensive analysis of this population. In this large real-world, retrospective study, adults with NVAF at high risk for GIB who received apixaban, dabigatran, and rivaroxaban had lower risks of MB, ICH, and stroke/SE versus those who received VKAs. Apixaban was associated with lower risk of GIB versus VKAs, while rivaroxaban and dabigatran had similar risks to VKAs. Results in key subgroups, such as standard dose and low dose populations, were generally consistent with findings from the main analysis. The percentage of patients in this study receiving a reduced dose of anticoagulants (42.8%) is also of note; this is higher than what has been reported in other recent studies in Europe and North America, which range from 20–30% [20]. However, given the high risk of bleeding for this population, physician may have been inclined to prescribed lower doses to manage this risk.

Given the differences in terms of efficacy/effectiveness and safety between individual DOACs shown in clinical trials and observational studies, and the risk of MB and stroke in particular [21,22], DOACs should be evaluated separately and not as a group. Large phase 3 studies of apixaban, rivaroxaban, and dabigatran have consistently shown reduced risk of stroke and ICH compared to VKAs, particularly warfarin [23]. A recent retrospective analysis evaluating a similar patient population in the US found that DOACs were associated with lower rates of MB and stroke/SE versus warfarin in NVAF patients with high GIB risk [8]. Indeed, real world data in NVAF patients with diabetes show less MB associated with apixaban compared to rivaroxaban [3]. In a study of European electronic health record data, the risk of GIB was increased by 48–67% in dabigatran users and 30–50% for rivaroxaban users compared to VKA users while apixaban was not associated with an increased risk of GIB [24]. Similarly, a network meta-analysis comparing DOACs with warfarin or enoxaparin found that risk of GIB was lower with standard dose apixaban compared to dabigatran and rivaroxaban [25].

The results of our analysis were generally consistent with those reported from the NAXOS study [22]; however, NAXOS utilized a broad population of NVAF patients both with and without risk factors for GIB (N = 321,501). In both studies, apixaban was associated with a lower risk of GIB versus all other oral ACs evaluated in NAXOS. Apixaban was also associated with a similar risk of stroke/SE compared to rivaroxaban in NAXOS, while in the current study this risk was reduced with apixaban. Similarly, in a recent population-based cohort study of new DOAC users by Lau et al, apixaban use was associated with lower risk for GIB and similar rates of ischemic stroke or SE, ICH, and all-cause mortality compared with dabigatran, edoxaban, and rivaroxaban [6].

In the current study, the risk of MB, ICH, other bleeding, and hemorrhagic stroke was lower across all DOACs compared to VKAs. Apixaban was associated with a lower risk for GIB versus VKAs while dabigatran and rivaroxaban were associated with a similar risk compared to VKAs. Conversely, in the pivotal randomized clinical trials (RCT) evaluating DOACs versus warfarin, apixaban was the only DOAC to show similar risk of GIB compared to warfarin [21], whereas dabigatran and rivaroxaban showed increased risk [26,27]. The differences observed between the current study and the pivot trials could be related to controlled nature of RCTs resulting in differences in quality of VKA monitoring and medication adherence compared to a ‘real- world’ setting [28]. In addition, the mechanism of action, rates of absorption, and dosing schedules for DOACs varies widely between agents and may result in topical and/or systemic damage to the gastrointestinal system, leading to varying rates of bleeds [12,29].

Results of randomized clinical trials of DOACs have showed consistent benefit versus VKAs; however, these studies include broad patient populations and may not stratify participants based on GIB risk. In the ARISTOTLE study, apixaban was shown to be superior to warfarin for key outcomes of stroke/SE and MB events among patients with NVAF and ≥1 prior risk factor for stroke; risk of GIB was similar between treatment groups [21]. In the ROCKET AF study, major GIB was more common in patients receiving rivaroxaban than those receiving warfarin (*p*<0.001) regardless of prior risk factors [27]. Similarly, in the RE-LY study, there was a significantly higher rate of major GIB with 150-mg dabigatran versus warfarin [26]. These results are in contrast with those reported in the current study, which showed similar risk of GIB with both rivaroxaban versus VKAs and dabigatran versus VKAs. In the present study, risk of ICH was less frequent with dabigatran compared to apixaban and rivaroxaban. Gómez-Outes et al reported results of a network meta-analysis of randomized clinical trials which showed reduced risk of ICH with all three DOACs versus warfarin [30]. A recent real-world analysis of US patients reported similar risk of ICH between dabigatran and apixaban as well as dabigatran and rivaroxaban [8]. These differences in risk between DOACs may be due to differences in each study population’s disease severity, age, and other clinical characteristics that can contribute to bleeding risk.

Selecting the appropriate dosage for DOACs is an important consideration to minimize bleeding risk per recent guidelines [1,14]. Dose reduction criteria include weight ≤60 kg, age ≥80 years, body weight ≤60 kg, and impaired kidney function as measured by creatinine clearance or serum creatinine [4]. In the current analysis, standard and reduced doses of all DOACs were associated with a lower risk of ICH, other bleeds, and stroke/SE versus VKAs.

### Limitations

This study is limited by several factors. Given the study’s retrospective design, claims were used to determine risk factors for GIB which may be misclassified due to lack of information or data entry errors. Our results, however, are strengthened by PS matching to align the treatment cohorts using demographic and clinical characteristics. Results of sensitivity analyses were also similar to the main findings of the analysis. Because large datasets were used, very small departures from proportional hazards can be detected without an impact on the overall results; this was confirmed by AFT analyses, which were similar to the main Cox proportional hazards analysis. Our results are limited to clinical practice in France which may not be generalized to other countries and NVAF patient populations; however, the study uses the SNDS database which covers almost the entire of the French population, giving rich information on a range of data points as well as statistical power for robust comparative evidence. Additionally, using a single database that covers most of the patient population minimizes selection bias. While apixaban was prescribed to more than half (51.6%) of the included patients, which may reflect the physician’s preferences in real-world practice, PS matching was performed in order to minimize potential bias. However, despite PS matching residual confounding is still possible. Finally, patients with probable AF (prescription of any anti-arrhythmic drugs dispensed concomitantly with VKAs or DOACs) were included in the main study population as a proxy for outpatient data, as these patients have less severe disease burden compared to those with ICD-10 diagnosis codes for hospitalization. This method may not be as sensitive as using diagnosis codes; however, results of sensitivity analyses excluding this subgroup are consistent with results in the overall study population.

## Conclusions

DOACs were associated with improved safety and effectiveness from MB and stroke/SE, respectively, when compared to VKAs among patients with NVAF at high risk for GIB. Apixaban was associated with lower MB and GIB risk versus other DOACs. For stroke/SE, apixaban was associated with reduced risk compared to rivaroxaban and similar risk compared to dabigatran.

## Supporting information

S1 Table ICD-10 codes used to identify safety and effectiveness outcomes. (DOCX)

S2 Table Baseline characteristics after PS matching for the standard and reduced dose subgroups–DOAC–VKA comparisons. (DOCX)

S3 Table Baseline characteristics after PS matching for the standard and reduced dose subgroups–DOAC–DOAC comparisons. (DOCX)

S4 Table Demographic and clinical characteristics prior to PS matching. (DOCX)

S5 Table Outcome rates by cohort prior to PS matching. (DOCX)

S6 Table Estimated relative acceleration factors and 95% CI from the AFT analysis (PS matched population). (DOCX)

S1 Fig Kaplan-Meier curves for ICH. Apixaban versus VKAs (A), dabigatran versus VKAs (B), and rivaroxaban versus VKAs (C), rivaroxaban versus apixaban (D), dabigatran versus apixaban (E), and dabigatran versus rivaroxaban (F).
(DOCX)

S2 Fig Kaplan-Meier curves for other bleeding. Apixaban versus VKAs (A), dabigatran versus VKAs (B), and rivaroxaban versus VKAs (C), rivaroxaban versus apixaban (D), dabigatran versus apixaban (E), and dabigatran versus rivaroxaban (F).
(DOCX)

S3 Fig Kaplan-Meier curves for stroke. Apixaban versus VKAs (A), dabigatran versus VKAs (B), and rivaroxaban versus VKAs (C), rivaroxaban versus apixaban (D), dabigatran versus apixaban (E), and dabigatran versus rivaroxaban (F).
(DOCX)

S4 Fig Kaplan-Meier curves for SE. Apixaban versus VKAs (A), dabigatran versus VKAs (B), and rivaroxaban versus VKAs (C), rivaroxaban versus apixaban (D), dabigatran versus apixaban (E), and dabigatran versus rivaroxaban (F).
(DOCX)

S5 Fig Kaplan-Meier curves for hemorrhagic stroke. Apixaban versus VKAs (A), dabigatran versus VKAs (B), and rivaroxaban versus VKAs (C), rivaroxaban versus apixaban (D), dabigatran versus apixaban (E), and dabigatran versus rivaroxaban (F).
(DOCX)

S6 Fig Kaplan-Meier curves for ischemic stroke. Apixaban versus VKAs (A), dabigatran versus VKAs (B), and rivaroxaban versus VKAs (C), rivaroxaban versus apixaban (D), dabigatran versus apixaban (E), and dabigatran versus rivaroxaban (F).
(DOCX)

S7 Fig PS-matched hazard ratios for sensitivity analyses excluding probable AF. Apixaban versus VKAs (A), dabigatran versus VKAs (B), rivaroxaban versus VKAs (C), apixaban versus dabigatran (D), dabigatran versus rivaroxaban (E), and apixaban versus rivaroxaban (F).
(DOCX)
