# Supplementary material for: Comparative safety and effectiveness of oral anticoagulants in patients with non-valvular atrial fibrillation and high risk of gastrointestinal bleeding: A nationwide French cohort study
Source: PLoS One. 2024 Nov 15;19(11):e0310322. doi: 10.1371/journal.pone.0310322 (PMC11567525; doi:10.1371/journal.pone.0310322)
Supplement: S2 Table — (DOCX) [file pone.0310322.s002.docx]

**Supplementary Table 2**. Baseline characteristics after PS matching for the standard and reduced dose subgroups – DOAC–VKA comparisons

|  | | **Standard dose** | | **Reduced dose** | | **Standard dose** | | **Reduced dose** | | **Standard dose** | | **Reduced dose** | |
| --- | --- | --- | --- | --- | --- | --- | --- | --- | --- | --- | --- | --- | --- |
| **Characteristic** | | **Apixaban (n = 35,600)** | **VKAs (n = 35,600)** | **Apixaban (n = 40,398)** | **VKAs (n = 40,398)** | **Rivaroxaban (n = 27,611)** | **VKAs (n = 27,611)** | **Rivaroxaban (n = 28,943)** | **VKAs (n = 28,943)** | **Dabigatran (n = 5,698)** | **VKAs (n = 5,698)** | **Dabigatran**  **(n = 10,765)** | **VKAs (n = 10,765)** |
| **Atrial fibrillation identification setting** | Inpatient claim with I48 code | 26887 (75.53%) | 26148 (73.45%) | 30376 (75.19%) | 30565 (75.66%) | 19286 (69.85%) | 19017 (68.87%) | 18800 (64.96%) | 19444 (67.18%) | 2888 (50.68%) | 2841 (49.86%) | 6364 (59.12%) | 6220 (57.78%) |
|  | LTR registration with I48 code | 1946 (5.47%) | 1974 (5.54%) | 2175 (5.38%) | 2018 (5%) | 1869 (6.77%) | 1844 (6.68%) | 2274 (7.86%) | 1991 (6.88%) | 805 (14.13%) | 759 (13.32%) | 1098 (10.2%) | 1114 (10.35%) |
|  | Use of anti-arrhythmic drugs | 6767 (19.01%) | 7478 (21.01%) | 7847 (19.42%) | 7815 (19.35%) | 6456 (23.38%) | 6750 (24.45%) | 7869 (27.19%) | 7508 (25.94%) | 2005 (35.19%) | 2098 (36.82%) | 3303 (30.68%) | 3431 (31.87%) |
| **Age at index date (years)** | | 78.7 [10.2] | 79.7 [10.1] | 83.5 [8.8] | 82.9 [9.1] | 77.1 [10.3] | 78.1 [10] | 81.6 [9.3] | 81.6 [9.6] | 70.8 [9.3] | 71.4 [9.5] | 81.5 [8.4] | 81.6 [8.5] |
| **Age groups at index date** | 18-54 years | 742 (2.08%) | 896 (2.52%) | 305 (0.75%) | 370 (0.92%) | 885 (3.21%) | 710 (2.57%) | 403 (1.39%) | 428 (1.48%) | 285 (5%) | 290 (5.09%) | 107 (0.99%) | 103 (0.96%) |
|  | 55-64 years | 2174 (6.11%) | 2511 (7.05%) | 1133 (2.8%) | 1278 (3.16%) | 2308 (8.36%) | 2030 (7.35%) | 1183 (4.09%) | 1235 (4.27%) | 911 (15.99%) | 849 (14.9%) | 356 (3.31%) | 342 (3.18%) |
|  | 65-74 years | 6162 (17.31%) | 6810 (19.13%) | 4297 (10.64%) | 4657 (11.53%) | 6086 (22.04%) | 5527 (20.02%) | 3739 (12.92%) | 3790 (13.09%) | 2252 (39.52%) | 2182 (38.29%) | 1267 (11.77%) | 1237 (11.49%) |
|  | 75-79 years | 5617 (15.78%) | 5820 (16.35%) | 4859 (12.03%) | 5271 (13.05%) | 4931 (17.86%) | 4983 (18.05%) | 4238 (14.64%) | 4123 (14.25%) | 1450 (25.45%) | 1560 (27.38%) | 1732 (16.09%) | 1760 (16.35%) |
|  | 80-84 years | 7822 (21.97%) | 7588 (21.31%) | 8759 (21.68%) | 8658 (21.43%) | 6543 (23.7%) | 6277 (22.73%) | 6515 (22.51%) | 6836 (23.62%) | 533 (9.35%) | 547 (9.6%) | 3060 (28.43%) | 3083 (28.64%) |
|  | 85-89 years | 8142 (22.87%) | 7774 (21.84%) | 10428 (25.81%) | 10802 (26.74%) | 5650 (20.46%) | 5178 (18.75%) | 7264 (25.1%) | 7191 (24.85%) | 221 (3.88%) | 198 (3.47%) | 2772 (25.75%) | 2737 (25.42%) |
|  | 90-94 years | 4220 (11.85%) | 3580 (10.06%) | 7360 (18.22%) | 7740 (19.16%) | 1785 (6.46%) | 1604 (5.81%) | 4461 (15.41%) | 4267 (14.74%) | 49 (0.86%) | 44 (0.77%) | 1216 (11.3%) | 1214 (11.28%) |
|  | ≥95 years | 721 (2.03%) | 621 (1.74%) | 2275 (5.63%) | 2604 (6.45%) | 383 (1.39%) | 342 (1.24%) | 1127 (3.89%) | 1086 (3.75%) | 14 (0.25%) | 11 (0.19%) | 275 (2.55%) | 269 (2.5%) |
| **Sex** | Male | 17816 (50.0%) | 18227 (51.2%) | 17341 (42.9%) | 17877 (44.2%) | 14506 (52.5%) | 14351 (52.0%) | 13378 (46.2%) | 13449 (46.5%) | 3572 (62.7%) | 3477 (61.0%) | 4905 (45.6%) | 4930 (45.8%) |
|  | Female | 17784 (49.96%) | 17373 (48.8%) | 23057 (57.07%) | 22521 (55.75%) | 13105 (47.46%) | 13260 (48.02%) | 15565 (53.78%) | 15494 (53.53%) | 2126 (37.31%) | 2221 (38.98%) | 5860 (54.44%) | 5835 (54.2%) |
| **GIB risk factors** | Age ≥75 years | 26522 (74.5%) | 25383 (71.3%) | 34663 (85.8%) | 34093 (84.39%) | 18332 (66.39%) | 19344 (70.06%) | 23618 (81.6%) | 23490 (81.16%) | 2250 (39.49%) | 2377 (41.72%) | 9035 (83.93%) | 9083 (84.38%) |
|  | HAS-BLED score, mean | 2.9 [1] | 2.9 [1] | 3 [1] | 3 [1.1] | 2.7 [1] | 2.7 [1] | 2.8 [1] | 2.8 [1] | 2.5 [1] | 2.5 [1] | 2.7 [1] | 2.7 [1] |
|  | 0 | 108 (0.3%) | 196 (0.55%) | 69 (0.17%) | 74 (0.18%) | 156 (0.56%) | 108 (0.39%) | 68 (0.23%) | 84 (0.29%) | 91 (1.6%) | 83 (1.46%) | 23 (0.21%) | 18 (0.17%) |
|  | 1 | 2424 (6.81%) | 2337 (6.56%) | 2162 (5.35%) | 2333 (5.78%) | 2296 (8.32%) | 2273 (8.23%) | 2295 (7.93%) | 2239 (7.74%) | 755 (13.25%) | 720 (12.64%) | 999 (9.28%) | 1024 (9.51%) |
|  | 2 | 9966 (27.99%) | 9680 (27.19%) | 9825 (24.32%) | 10225 (25.31%) | 8573 (31.05%) | 8563 (31.01%) | 8549 (29.54%) | 8765 (30.28%) | 1864 (32.71%) | 1862 (32.68%) | 3398 (31.57%) | 3418 (31.75%) |
|  | ≥3 | 23102 (64.89%) | 23387 (65.69%) | 28342 (70.16%) | 27766 (68.73%) | 16586 (60.07%) | 16667 (60.36%) | 18031 (62.3%) | 17855 (61.69%) | 2988 (52.44%) | 3033 (53.23%) | 6345 (58.94%) | 6305 (58.57%) |
|  | Prior medications | 21834 (61.33%) | 20942 (58.83%) | 24021 (59.46%) | 23175 (57.37%) | 17275 (62.57%) | 16499 (59.76%) | 18103 (62.55%) | 17635 (60.93%) | 4012 (70.41%) | 3926 (68.9%) | 6131 (56.95%) | 6121 (56.86%) |
|  | Renal impairment | 1916 (5.38%) | 2545 (7.15%) | 4914 (12.16%) | 5110 (12.65%) | 740 (2.68%) | 1056 (3.82%) | 1912 (6.61%) | 1980 (6.84%) | 61 (1.07%) | 57 (1%) | 334 (3.1%) | 361 (3.35%) |
|  | Prior GI condition | 3483 (9.78%) | 2985 (8.38%) | 3538 (8.76%) | 3402 (8.42%) | 2529 (9.16%) | 2234 (8.09%) | 2138 (7.39%) | 2086 (7.21%) | 507 (8.9%) | 470 (8.25%) | 866 (8.04%) | 818 (7.6%) |
| **Number of GIB risk factors** | 1 | 11208 (31.48%) | 11666 (32.77%) | 9852 (24.39%) | 11104 (27.49%) | 10111 (36.62%) | 10250 (37.12%) | 9032 (31.21%) | 9604 (33.18%) | 2743 (48.14%) | 2695 (47.3%) | 3764 (34.97%) | 3816 (35.45%) |
|  | 2 | 10168 (28.56%) | 9416 (26.45%) | 10147 (25.12%) | 9614 (23.8%) | 8045 (29.14%) | 7554 (27.36%) | 6752 (23.33%) | 6438 (22.24%) | 1887 (33.12%) | 1911 (33.54%) | 2539 (23.59%) | 2482 (23.06%) |
|  | 3 | 12507 (35.13%) | 12555 (35.27%) | 16509 (40.87%) | 15735 (38.95%) | 8586 (31.1%) | 8815 (31.93%) | 11472 (39.64%) | 11110 (38.39%) | 972 (17.06%) | 1023 (17.95%) | 4003 (37.19%) | 3978 (36.95%) |
|  | 4 | 1647 (4.63%) | 1882 (5.29%) | 3645 (9.02%) | 3716 (9.2%) | 842 (3.05%) | 963 (3.49%) | 1585 (5.48%) | 1719 (5.94%) | 95 (1.67%) | 68 (1.19%) | 435 (4.04%) | 471 (4.38%) |
|  | 5 | 70 (0.2%) | 81 (0.23%) | 245 (0.61%) | 229 (0.57%) | 27 (0.1%) | 29 (0.11%) | 102 (0.35%) | 72 (0.25%) | 1 (0.02%) | 1 (0.02%) | 24 (0.22%) | 18 (0.17%) |
| **Charlson Comorbidity Index score** | 0 | 6397 (17.97%) | 7382 (20.74%) | 7005 (17.34%) | 7453 (18.45%) | 6592 (23.87%) | 6698 (24.26%) | 7555 (26.1%) | 7182 (24.81%) | 2159 (37.89%) | 2142 (37.59%) | 3617 (33.6%) | 3578 (33.24%) |
|  | 1 or 2 | 16553 (46.5%) | 16005 (44.96%) | 17570 (43.49%) | 17065 (42.24%) | 13463 (48.76%) | 13123 (47.53%) | 13401 (46.3%) | 13567 (46.87%) | 2398 (42.08%) | 2418 (42.44%) | 4573 (42.48%) | 4584 (42.58%) |
|  | 3 or 4 | 8185 (22.99%) | 7935 (22.29%) | 9985 (24.72%) | 10044 (24.86%) | 5084 (18.41%) | 5310 (19.23%) | 5344 (18.46%) | 5539 (19.14%) | 840 (14.74%) | 828 (14.53%) | 1720 (15.98%) | 1783 (16.56%) |
|  | ≥5 | 4465 (12.54%) | 4278 (12.02%) | 5838 (14.45%) | 5836 (14.45%) | 2472 (8.95%) | 2480 (8.98%) | 2643 (9.13%) | 2655 (9.17%) | 301 (5.28%) | 310 (5.44%) | 855 (7.94%) | 820 (7.62%) |
| **Additional comorbidities** | Myocardial infarction | 3407 (9.57%) | 3413 (9.59%) | 4600 (11.39%) | 4389 (10.86%) | 2194 (7.95%) | 2277 (8.25%) | 3429 (11.85%) | 3372 (11.65%) | 243 (4.26%) | 240 (4.21%) | 643 (5.97%) | 626 (5.82%) |
|  | Congestive heart failure | 13753 (38.63%) | 13778 (38.7%) | 17175 (42.51%) | 17674 (43.75%) | 9052 (32.78%) | 9338 (33.82%) | 10006 (34.57%) | 10301 (35.59%) | 951 (16.69%) | 934 (16.39%) | 2671 (24.81%) | 2641 (24.53%) |
|  | Peripheral vascular disease | 4262 (11.97%) | 4075 (11.45%) | 4828 (11.95%) | 4730 (11.71%) | 2796 (10.13%) | 2805 (10.16%) | 2692 (9.3%) | 2730 (9.43%) | 349 (6.12%) | 363 (6.37%) | 761 (7.07%) | 783 (7.27%) |
|  | Cerebrovascular disease | 7330 (20.59%) | 6621 (18.6%) | 7758 (19.2%) | 7305 (18.08%) | 4425 (16.03%) | 4390 (15.9%) | 3713 (12.83%) | 3788 (13.09%) | 1120 (19.66%) | 1114 (19.55%) | 2025 (18.81%) | 1941 (18.03%) |
|  | Dementia | 2997 (8.42%) | 3104 (8.72%) | 5399 (13.36%) | 4853 (12.01%) | 1922 (6.96%) | 2092 (7.58%) | 2865 (9.9%) | 2950 (10.19%) | 87 (1.53%) | 83 (1.46%) | 760 (7.06%) | 713 (6.62%) |
|  | Chronic pulmonary disease | 8720 (24.49%) | 7944 (22.31%) | 9420 (23.32%) | 8870 (21.96%) | 6589 (23.86%) | 6028 (21.83%) | 6249 (21.59%) | 6127 (21.17%) | 1053 (18.48%) | 1077 (18.9%) | 2080 (19.32%) | 2107 (19.57%) |
|  | Connective tissue disease | 698 (1.96%) | 625 (1.76%) | 822 (2.03%) | 740 (1.83%) | 453 (1.64%) | 451 (1.63%) | 415 (1.43%) | 424 (1.46%) | 66 (1.16%) | 58 (1.02%) | 140 (1.3%) | 130 (1.21%) |
|  | Ulcer disease | 479 (1.35%) | 482 (1.35%) | 577 (1.43%) | 574 (1.42%) | 303 (1.1%) | 293 (1.06%) | 269 (0.93%) | 286 (0.99%) | 41 (0.72%) | 44 (0.77%) | 118 (1.1%) | 130 (1.21%) |
|  | Mild liver disease | 1019 (2.86%) | 945 (2.65%) | 667 (1.65%) | 701 (1.74%) | 707 (2.56%) | 724 (2.62%) | 362 (1.25%) | 391 (1.35%) | 81 (1.42%) | 69 (1.21%) | 137 (1.27%) | 141 (1.31%) |
|  | Diabetes | 8820 (24.78%) | 8386 (23.56%) | 8779 (21.73%) | 8905 (22.04%) | 6441 (23.33%) | 6303 (22.83%) | 5993 (20.71%) | 6097 (21.07%) | 1166 (20.46%) | 1197 (21.01%) | 1907 (17.71%) | 1908 (17.72%) |
|  | Diabetes with end-organ damage | 998 (2.8%) | 1064 (2.99%) | 1318 (3.26%) | 1433 (3.55%) | 554 (2.01%) | 613 (2.22%) | 626 (2.16%) | 664 (2.29%) | 66 (1.16%) | 60 (1.05%) | 155 (1.44%) | 145 (1.35%) |
|  | Hemiplegia | 3299 (9.27%) | 2848 (8%) | 3014 (7.46%) | 2977 (7.37%) | 1812 (6.56%) | 1818 (6.58%) | 1226 (4.24%) | 1252 (4.33%) | 522 (9.16%) | 505 (8.86%) | 915 (8.5%) | 886 (8.23%) |
|  | Moderate or severe renal disease | 3708 (10.42%) | 4253 (11.95%) | 7741 (19.16%) | 8291 (20.52%) | 1522 (5.51%) | 1868 (6.77%) | 3138 (10.84%) | 3331 (11.51%) | 139 (2.44%) | 159 (2.79%) | 590 (5.48%) | 693 (6.44%) |
|  | Any tumor (except for malignant neoplasm of skin) | 3838 (10.78%) | 3498 (9.83%) | 4131 (10.23%) | 3925 (9.72%) | 2800 (10.14%) | 2660 (9.63%) | 2534 (8.76%) | 2561 (8.85%) | 423 (7.42%) | 447 (7.84%) | 934 (8.68%) | 899 (8.35%) |
|  | Metastatic solid tumor | 861 (2.42%) | 747 (2.1%) | 802 (1.99%) | 772 (1.91%) | 654 (2.37%) | 615 (2.23%) | 506 (1.75%) | 527 (1.82%) | 67 (1.18%) | 82 (1.44%) | 214 (1.99%) | 201 (1.87%) |
|  | HIV/ AIDS | 64 (0.18%) | 62 (0.17%) | 25 (0.06%) | 33 (0.08%) | 42 (0.15%) | 52 (0.19%) | 25 (0.09%) | 23 (0.08%) | 5 (0.09%) | 6 (0.11%) | 7 (0.07%) | 8 (0.07%) |
|  | Moderate or severe liver disease | 227 (0.64%) | 254 (0.71%) | 159 (0.39%) | 169 (0.42%) | 164 (0.59%) | 190 (0.69%) | 62 (0.21%) | 80 (0.28%) | 12 (0.21%) | 7 (0.12%) | 43 (0.4%) | 32 (0.3%) |
| **CHA_2_DS_2_-VASc score** | Mean (SD) | 4.2 [1.5] | 4.2 [1.4] | 4.5 [1.4] | 4.5 [1.4] | 3.9 [1.5] | 4 [1.4] | 4.1 [1.4] | 4.1 [1.4] | 3.2 [1.5] | 3.2 [1.5] | 4.1 [1.4] | 4 [1.3] |
|  | 0 | 252 (0.71%) | 191 (0.54%) | 123 (0.3%) | 145 (0.36%) | 297 (1.08%) | 189 (0.68%) | 154 (0.53%) | 131 (0.45%) | 197 (3.46%) | 160 (2.81%) | 59 (0.55%) | 49 (0.46%) |
|  | 1 | 1022 (2.87%) | 823 (2.31%) | 485 (1.2%) | 598 (1.48%) | 1049 (3.8%) | 810 (2.93%) | 617 (2.13%) | 576 (1.99%) | 578 (10.14%) | 539 (9.46%) | 193 (1.79%) | 198 (1.84%) |
|  | 2–3 | 9645 (27.09%) | 9712 (27.28%) | 8448 (20.92%) | 8789 (21.76%) | 9055 (32.79%) | 8862 (32.10%) | 8312 (28.72%) | 7995 (27.63%) | 2595 (45.55%) | 2714 (47.63%) | 3393 (31.52%) | 3445 (32.00%) |
|  | ≥4 | 24681 (69.33%) | 24874 (69.87%) | 31342 (77.58%) | 30866 (76.4%) | 17210 (62.33%) | 17750 (64.29%) | 19924 (68.84%) | 20177 (69.71%) | 2328 (40.86%) | 2285 (40.1%) | 7120 (66.14%) | 7073 (65.7%) |
| **Concomitant treatment** | Antiplatelets | 17539 (49.27%) | 17490 (49.13%) | 20048 (49.63%) | 19662 (48.67%) | 13530 (49%) | 13538 (49.03%) | 15321 (52.94%) | 14929 (51.58%) | 2831 (49.68%) | 2873 (50.42%) | 4998 (46.43%) | 5008 (46.52%) |
|  | Aromatase inhibitors | 294 (0.83%) | 250 (0.7%) | 355 (0.88%) | 314 (0.78%) | 237 (0.86%) | 200 (0.72%) | 237 (0.82%) | 221 (0.76%) | 36 (0.63%) | 38 (0.67%) | 69 (0.64%) | 64 (0.59%) |
|  | NSAIDs | 3032 (8.52%) | 2374 (6.67%) | 2983 (7.38%) | 2418 (5.99%) | 2792 (10.11%) | 2097 (7.59%) | 2320 (8.02%) | 2050 (7.08%) | 966 (16.95%) | 879 (15.43%) | 908 (8.43%) | 861 (8%) |
|  | H2-receptor antagonists | 190 (0.53%) | 169 (0.47%) | 211 (0.52%) | 188 (0.47%) | 142 (0.51%) | 125 (0.45%) | 126 (0.44%) | 129 (0.45%) | 26 (0.46%) | 27 (0.47%) | 44 (0.41%) | 50 (0.46%) |
|  | Prostaglandins | 799 (2.24%) | 496 (1.39%) | 987 (2.44%) | 536 (1.33%) | 715 (2.59%) | 383 (1.39%) | 576 (1.99%) | 379 (1.31%) | 166 (2.91%) | 106 (1.86%) | 190 (1.76%) | 129 (1.2%) |
|  | Proton pump inhibitors | 19028 (53.45%) | 18460 (51.85%) | 21808 (53.98%) | 21303 (52.73%) | 14024 (50.79%) | 13681 (49.55%) | 14386 (49.7%) | 14544 (50.25%) | 2369 (41.58%) | 2406 (42.23%) | 5062 (47.02%) | 5083 (47.22%) |
|  | Anticonvulsant strong inhibitor of hepatic enzymes | 311 (0.87%) | 283 (0.79%) | 308 (0.76%) | 300 (0.74%) | 242 (0.88%) | 233 (0.84%) | 178 (0.62%) | 184 (0.64%) | 31 (0.54%) | 30 (0.53%) | 62 (0.58%) | 63 (0.59%) |
|  | HIV protease inhibitors | 161 (0.45%) | 119 (0.33%) | 126 (0.31%) | 117 (0.29%) | 130 (0.47%) | 94 (0.34%) | 81 (0.28%) | 76 (0.26%) | 28 (0.49%) | 32 (0.56%) | 27 (0.25%) | 17 (0.16%) |
|  | Strong inhibitors of both CYP3A4 and P-gp | 589 (1.65%) | 450 (1.26%) | 618 (1.53%) | 494 (1.22%) | 438 (1.59%) | 335 (1.21%) | 391 (1.35%) | 354 (1.22%) | 105 (1.84%) | 116 (2.04%) | 152 (1.41%) | 147 (1.37%) |
|  | Statins | 6653 (18.69%) | 6110 (17.16%) | 6499 (16.09%) | 6416 (15.88%) | 5126 (18.57%) | 4808 (17.41%) | 4817 (16.64%) | 4728 (16.34%) | 961 (16.87%) | 1014 (17.8%) | 1811 (16.82%) | 1786 (16.59%) |
|  | Selective estrogen receptor modulators | 71 (0.2%) | 67 (0.19%) | 84 (0.21%) | 70 (0.17%) | 62 (0.22%) | 53 (0.19%) | 57 (0.2%) | 53 (0.18%) | 11 (0.19%) | 10 (0.18%) | 21 (0.2%) | 16 (0.15%) |
|  | Selective serotonin reuptake inhibitors | 3708 (10.42%) | 3251 (9.13%) | 4204 (10.41%) | 3853 (9.54%) | 2453 (8.88%) | 2337 (8.46%) | 2531 (8.74%) | 2532 (8.75%) | 383 (6.72%) | 371 (6.51%) | 924 (8.58%) | 896 (8.32%) |
|  | Hormones | 1103 (3.1%) | 764 (2.15%) | 1357 (3.36%) | 815 (2.02%) | 1013 (3.67%) | 589 (2.13%) | 885 (3.06%) | 607 (2.1%) | 245 (4.3%) | 181 (3.18%) | 300 (2.79%) | 230 (2.14%) |
|  | Erythropoesis stimulating agents | 246 (0.69%) | 266 (0.75%) | 516 (1.28%) | 554 (1.37%) | 153 (0.55%) | 188 (0.68%) | 165 (0.57%) | 186 (0.64%) | 11 (0.19%) | 10 (0.18%) | 35 (0.33%) | 39 (0.36%) |
|  | Beta blockers | 21813 (61.27%) | 21855 (61.39%) | 24057 (59.55%) | 24416 (60.44%) | 16533 (59.88%) | 16616 (60.18%) | 16909 (58.42%) | 17142 (59.23%) | 3297 (57.86%) | 3279 (57.55%) | 5927 (55.06%) | 5882 (54.64%) |
|  | Antiarrhythmic agents | 17266 (48.5%) | 17817 (50.05%) | 19796 (49%) | 19542 (48.37%) | 14464 (52.38%) | 14711 (53.28%) | 16417 (56.72%) | 15961 (55.15%) | 3631 (63.72%) | 3657 (64.18%) | 6300 (58.52%) | 6381 (59.28%) |

Scores (Charlson Comorbidity Index, HAS-BLED and CHA_2_DS_2_-VASc) were not included in the PS modelling as their components are singularly included, but were used as indicator for evaluating the fitness of the matching. AIDS, acquired immunodeficiency syndrome; CYP3A4, cytochrome P450 3A4; DOAC, direct oral anticoagulant; GIB, gastrointestinal bleed; HIV, human immunodeficiency virus; LTR, long-term recurrence; NSAID, nonsteroidal anti-inflammatory drug; P-gp, P-glycoprotein; PS, propensity score; SD, standard deviation; VKA, vitamin K antagonist.
