# Supplementary material for: Comparative safety and effectiveness of oral anticoagulants in patients with non-valvular atrial fibrillation and high risk of gastrointestinal bleeding: A nationwide French cohort study
Source: PLoS One. 2024 Nov 15;19(11):e0310322. doi: 10.1371/journal.pone.0310322 (PMC11567525; doi:10.1371/journal.pone.0310322)
Supplement: S3 Table — (DOCX) [file pone.0310322.s003.docx]

**Supplementary Table 3.** Baseline characteristics after PS matching for the standard and reduced dose subgroups – DOAC–DOAC comparisons

|  | | | **Standard dose** | | | | | **Reduced dose** | | | | | **Standard dose** | | | | | **Reduced dose** | | | | | **Standard dose** | | | | | **Reduced dose** | | | |
| --- | --- | --- | --- | --- | --- | --- | --- | --- | --- | --- | --- | --- | --- | --- | --- | --- | --- | --- | --- | --- | --- | --- | --- | --- | --- | --- | --- | --- | --- | --- | --- |
| **Characteristic** | | | **Apixaban (n = 5,766)** | | **Dabigatran (n = 5,699)** | | **Apixaban (n = 10,698)** | | | **Dabigatran (n = 10,765)** | | **Rivaroxaban (n = 5,736)** | | | **Dabigatran (n = 5,699)** | | **Rivaroxaban (n = 10,723)** | | | **Dabigatran (n = 10,760)** | | **Apixaban (n = 56,700)** | | | **Rivaroxaban (n = 56,125)** | | **Apixaban (n = 31,714)** | | | **Rivaroxaban (n = 32,289)** | |
| **Atrial fibrillation identification setting** | Inpatient claim with I48 code | 3009 (52.19%) | | 2888 (50.68%) | | 5977 (55.87%) | | | 6365 (59.13%) | | 2944 (51.32%) | | | 2888 (50.68%) | | 5988 (55.84%) | | | 6359 (59.1%) | | 30559 (53.9%) | | | 30438 (54.23%) | | 18539 (58.46%) | | | 19182 (59.41%) | |  |
|  | LTR registration with I48 code | 811 (14.07%) | | 806 (14.14%) | | 1172 (10.96%) | | | 1098 (10.2%) | | 847 (14.77%) | | | 806 (14.14%) | | 1155 (10.77%) | | | 1098 (10.2%) | | 7196 (12.69%) | | | 7090 (12.63%) | | 2976 (9.38%) | | | 2870 (8.89%) | |  |
|  | Use of anti-arrhythmic drugs | 1946 (33.75%) | | 2005 (35.18%) | | 3549 (33.17%) | | | 3302 (30.67%) | | 1945 (33.91%) | | | 2005 (35.18%) | | 3580 (33.39%) | | | 3303 (30.7%) | | 18945 (33.41%) | | | 18597 (33.13%) | | 10199 (32.16%) | | | 10237 (31.7%) | |  |
| **Age at index date (years)** | | | 71.5 [9.5] | | 70.8 [9.3] | | 81.4 [8.6] | | | 81.5 [8.4] | | 72.3 [9.9] | | | 70.8 [9.3] | | 80.7 [8.8] | | | 81.5 [8.4] | | 73.5 [10.1] | | | 73.7 [10.7] | | 83 [8.5] | | | 81.7 [9] | |
| **Age groups at index date** | 18-54 years | 300 (5.2%) | | 285 (5%) | | 101 (0.94%) | | | 107 (0.99%) | | 254 (4.43%) | | | 285 (5%) | | 112 (1.04%) | | | 107 (0.99%) | | 2758 (4.86%) | | | 2938 (5.23%) | | 286 (0.9%) | | | 410 (1.27%) | |  |
|  | 55-64 years | 867 (15.04%) | | 911 (15.99%) | | 404 (3.78%) | | | 356 (3.31%) | | 807 (14.07%) | | | 911 (15.99%) | | 471 (4.39%) | | | 356 (3.31%) | | 7107 (12.53%) | | | 7050 (12.56%) | | 916 (2.89%) | | | 1222 (3.78%) | |  |
|  | 65-74 years | 2190 (37.98%) | | 2253 (39.53%) | | 1353 (12.65%) | | | 1266 (11.76%) | | 2018 (35.18%) | | | 2253 (39.53%) | | 1593 (14.86%) | | | 1263 (11.74%) | | 16764 (29.57%) | | | 15779 (28.11%) | | 2971 (9.37%) | | | 3996 (12.38%) | |  |
|  | 75-79 years | 1439 (24.96%) | | 1450 (25.44%) | | 1708 (15.97%) | | | 1732 (16.09%) | | 1400 (24.41%) | | | 1450 (25.44%) | | 1772 (16.53%) | | | 1730 (16.08%) | | 13611 (24.01%) | | | 12673 (22.58%) | | 4050 (12.77%) | | | 4783 (14.81%) | |  |
|  | 80-84 years | 619 (10.74%) | | 547 (9.6%) | | 2941 (27.49%) | | | 3084 (28.65%) | | 780 (13.6%) | | | 547 (9.6%) | | 2841 (26.49%) | | | 3084 (28.66%) | | 9949 (17.55%) | | | 10134 (18.06%) | | 8316 (26.22%) | | | 7878 (24.4%) | |  |
|  | 85-89 years | 292 (5.06%) | | 198 (3.47%) | | 2705 (25.29%) | | | 2737 (25.42%) | | 348 (6.07%) | | | 198 (3.47%) | | 2537 (23.66%) | | | 2737 (25.44%) | | 5055 (8.92%) | | | 5604 (9.98%) | | 8981 (28.32%) | | | 8379 (25.95%) | |  |
|  | 90-94 years | 51 (0.88%) | | 44 (0.77%) | | 1226 (11.46%) | | | 1214 (11.28%) | | 110 (1.92%) | | | 44 (0.77%) | | 1152 (10.74%) | | | 1214 (11.28%) | | 1254 (2.21%) | | | 1605 (2.86%) | | 4855 (15.31%) | | | 4511 (13.97%) | |  |
|  | ≥95 years | 8 (0.14%) | | 11 (0.19%) | | 260 (2.43%) | | | 269 (2.5%) | | 19 (0.33%) | | | 11 (0.19%) | | 245 (2.28%) | | | 269 (2.5%) | | 202 (0.36%) | | | 342 (0.61%) | | 1339 (4.22%) | | | 1110 (3.44%) | |  |
| **Sex** | Male | 3593 (62.3%) | | 3573 (62.7%) | | 4768 (44.6%) | | | 4905 (45.6%) | | 3488 (60.81%) | | | 3451 (60.55%) | | 4960 (46.3%) | | | 4902 (45.6%) | | 33629 (59.3%) | | | 32358 (57.7%) | | 13408 (42.3%) | | | 14818 (45.9%) | |  |
|  | Female | 2173 (37.69%) | | 2126 (37.3%) | | 5930 (55.43%) | | | 5860 (54.44%) | | 2248 (39.19%) | | | 2126 (37.3%) | | 5763 (53.74%) | | | 5858 (54.44%) | | 23071 (40.69%) | | | 23767 (42.35%) | | 18306 (57.72%) | | | 17471 (54.11%) | |  |
| **GIB risk factors** | Age ≥75 years | 2409 (41.78%) | | 2250 (39.48%) | | 8840 (82.63%) | | | 9036 (83.94%) | | 2657 (46.32%) | | | 2250 (39.48%) | | 8547 (79.71%) | | | 9034 (83.96%) | | 30071 (53.04%) | | | 30358 (54.09%) | | 27541 (86.84%) | | | 26661 (82.57%) | |  |
|  | HAS-BLED score, mean | 2.5 [1] | | 2.5 [1] | | 2.7 [1] | | | 2.7 [1] | | 2.5 [1] | | | 2.5 [1] | | 2.7 [1] | | | 2.7 [1] | | 2.5 [1] | | | 2.4 [1] | | 2.7 [1] | | | 2.7 [1] | |  |
|  | 0 | 104 (1.8%) | | 91 (1.6%) | | 30 (0.28%) | | | 23 (0.21%) | | 107 (1.87%) | | | 91 (1.6%) | | 29 (0.27%) | | | 23 (0.21%) | | 828 (1.46%) | | | 939 (1.67%) | | 60 (0.19%) | | | 72 (0.22%) | |  |
|  | 1 | 767 (13.3%) | | 755 (13.25%) | | 1059 (9.9%) | | | 999 (9.28%) | | 798 (13.91%) | | | 755 (13.25%) | | 1005 (9.37%) | | | 999 (9.28%) | | 7661 (13.51%) | | | 8023 (14.29%) | | 2808 (8.85%) | | | 2778 (8.6%) | |  |
|  | 2 | 1849 (32.07%) | | 1865 (32.73%) | | 3477 (32.5%) | | | 3398 (31.57%) | | 1908 (33.26%) | | | 1865 (32.73%) | | 3386 (31.58%) | | | 3397 (31.57%) | | 20034 (35.33%) | | | 19774 (35.23%) | | 10222 (32.23%) | | | 9982 (30.91%) | |  |
|  | ≥3 | 3046 (52.83%) | | 2988 (52.43%) | | 6132 (57.32%) | | | 6345 (58.94%) | | 2923 (50.96%) | | | 2988 (52.43%) | | 6303 (58.78%) | | | 6341 (58.93%) | | 28177 (49.69%) | | | 27389 (48.8%) | | 18624 (58.72%) | | | 19457 (60.26%) | |  |
|  | Prior medications | 4014 (69.61%) | | 4013 (70.42%) | | 6143 (57.42%) | | | 6132 (56.96%) | | 3862 (67.33%) | | | 4013 (70.42%) | | 6360 (59.31%) | | | 6129 (56.96%) | | 38311 (67.57%) | | | 37632 (67.05%) | | 18691 (58.94%) | | | 19936 (61.74%) | |  |
|  | Renal impairment | 52 (0.9%) | | 61 (1.07%) | | 325 (3.04%) | | | 334 (3.1%) | | 27 (0.47%) | | | 61 (1.07%) | | 336 (3.13%) | | | 334 (3.1%) | | 886 (1.56%) | | | 743 (1.32%) | | 1750 (5.52%) | | | 1912 (5.92%) | |  |
|  | Prior GI condition | 487 (8.45%) | | 507 (8.9%) | | 751 (7.02%) | | | 867 (8.05%) | | 513 (8.94%) | | | 507 (8.9%) | | 752 (7.01%) | | | 864 (8.03%) | | 4761 (8.4%) | | | 4813 (8.58%) | | 2077 (6.55%) | | | 2246 (6.96%) | |  |
| **Number of GIB risk factors** | 1 | 2721 (47.19%) | | 2744 (48.15%) | | 3874 (36.21%) | | | 3763 (34.96%) | | 2747 (47.89%) | | | 2744 (48.15%) | | 3829 (35.71%) | | | 3763 (34.97%) | | 26299 (46.38%) | | | 26304 (46.87%) | | 10769 (33.96%) | | | 10637 (32.94%) | |  |
|  | 2 | 1931 (33.49%) | | 1887 (33.11%) | | 2567 (24%) | | | 2539 (23.59%) | | 1812 (31.59%) | | | 1887 (33.11%) | | 2609 (24.33%) | | | 2536 (23.57%) | | 16434 (28.98%) | | | 15922 (28.37%) | | 6695 (21.11%) | | | 7208 (22.32%) | |  |
|  | 3 | 1035 (17.95%) | | 972 (17.06%) | | 3855 (36.03%) | | | 4003 (37.19%) | | 1098 (19.14%) | | | 972 (17.06%) | | 3903 (36.4%) | | | 4001 (37.18%) | | 12860 (22.68%) | | | 12836 (22.87%) | | 12564 (39.62%) | | | 12719 (39.39%) | |  |
|  | 4 | 75 (1.3%) | | 95 (1.67%) | | 392 (3.66%) | | | 436 (4.05%) | | 78 (1.36%) | | | 95 (1.67%) | | 368 (3.43%) | | | 436 (4.05%) | | 1076 (1.9%) | | | 1036 (1.85%) | | 1598 (5.04%) | | | 1623 (5.03%) | |  |
|  | 5 | 4 (0.07%) | | 1 (0.02%) | | 10 (0.09%) | | | 24 (0.22%) | | 1 (0.02%) | | | 1 (0.02%) | | 14 (0.13%) | | | 24 (0.22%) | | 31 (0.05%) | | | 27 (0.05%) | | 88 (0.28%) | | | 102 (0.32%) | |  |
| **Charlson Comorbidity Index score** | 0 | 2330 (40.41%) | | 2160 (37.9%) | | 3809 (35.6%) | | | 3617 (33.6%) | | 2325 (40.53%) | | | 2160 (37.9%) | | 3735 (34.83%) | | | 3617 (33.62%) | | 22447 (39.59%) | | | 22674 (40.4%) | | 10176 (32.09%) | | | 9868 (30.56%) | |  |
|  | 1 or 2 | 2371 (41.12%) | | 2398 (42.08%) | | 4487 (41.94%) | | | 4573 (42.48%) | | 2403 (41.89%) | | | 2398 (42.08%) | | 4457 (41.56%) | | | 4572 (42.49%) | | 24792 (43.72%) | | | 24282 (43.26%) | | 14141 (44.59%) | | | 14334 (44.39%) | |  |
|  | 3 or 4 | 805 (13.96%) | | 840 (14.74%) | | 1648 (15.4%) | | | 1719 (15.97%) | | 731 (12.74%) | | | 840 (14.74%) | | 1721 (16.05%) | | | 1718 (15.97%) | | 6626 (11.69%) | | | 6363 (11.34%) | | 4878 (15.38%) | | | 5425 (16.8%) | |  |
|  | ≥5 | 260 (4.51%) | | 301 (5.28%) | | 754 (7.05%) | | | 856 (7.95%) | | 277 (4.83%) | | | 301 (5.28%) | | 810 (7.55%) | | | 853 (7.93%) | | 2835 (5%) | | | 2806 (5%) | | 2519 (7.94%) | | | 2662 (8.24%) | |  |
| **Additional comorbidities** | Myocardial infarction | 190 (3.3%) | | 243 (4.26%) | | 673 (6.29%) | | | 643 (5.97%) | | 153 (2.67%) | | | 243 (4.26%) | | 732 (6.83%) | | | 643 (5.98%) | | 2993 (5.28%) | | | 2527 (4.5%) | | 3057 (9.64%) | | | 3617 (11.2%) | |  |
|  | Congestive heart failure | 888 (15.40%) | | 951 (16.69%) | | 2516 (23.52%) | | | 2671 (24.81%) | | 879 (15.32%) | | | 951 (16.69%) | | 2670 (24.90%) | | | 2670 (24.81%) | | 11889 (20.97%) | | | 11645 (20.75%) | | 9588 (30.23%) | | | 10122 (31.35%) | |  |
|  | Peripheral vascular disease | 290 (5.03%) | | 349 (6.12%) | | 711 (6.65%) | | | 761 (7.07%) | | 279 (4.86%) | | | 349 (6.12%) | | 749 (6.98%) | | | 760 (7.06%) | | 3499 (6.17%) | | | 3490 (6.22%) | | 2469 (7.79%) | | | 2748 (8.51%) | |  |
|  | Cerebrovascular disease | 1084 (18.8%) | | 1120 (19.65%) | | 1854 (17.33%) | | | 2025 (18.81%) | | 1112 (19.39%) | | | 1120 (19.65%) | | 1987 (18.53%) | | | 2021 (18.78%) | | 6307 (11.12%) | | | 6040 (10.76%) | | 3487 (11%) | | | 3760 (11.64%) | |  |
|  | Dementia | 95 (1.65%) | | 87 (1.53%) | | 716 (6.69%) | | | 760 (7.06%) | | 141 (2.46%) | | | 87 (1.53%) | | 660 (6.15%) | | | 759 (7.05%) | | 1743 (3.07%) | | | 2175 (3.88%) | | 3223 (10.16%) | | | 2940 (9.11%) | |  |
|  | Chronic pulmonary disease | 1014 (17.59%) | | 1053 (18.48%) | | 1967 (18.39%) | | | 2080 (19.32%) | | 1004 (17.5%) | | | 1053 (18.48%) | | 1981 (18.47%) | | | 2080 (19.33%) | | 11442 (20.18%) | | | 11270 (20.08%) | | 6318 (19.92%) | | | 6644 (20.58%) | |  |
|  | Connective tissue disease | 47 (0.82%) | | 66 (1.16%) | | 148 (1.38%) | | | 140 (1.3%) | | 67 (1.17%) | | | 66 (1.16%) | | 134 (1.25%) | | | 140 (1.3%) | | 645 (1.14%) | | | 702 (1.25%) | | 454 (1.43%) | | | 426 (1.32%) | |  |
|  | Ulcer disease | 39 (0.68%) | | 41 (0.72%) | | 97 (0.91%) | | | 118 (1.1%) | | 52 (0.91%) | | | 41 (0.72%) | | 99 (0.92%) | | | 117 (1.09%) | | 388 (0.68%) | | | 400 (0.71%) | | 259 (0.82%) | | | 273 (0.85%) | |  |
|  | Mild liver disease | 62 (1.08%) | | 81 (1.42%) | | 102 (0.95%) | | | 137 (1.27%) | | 68 (1.19%) | | | 81 (1.42%) | | 114 (1.06%) | | | 136 (1.26%) | | 845 (1.49%) | | | 870 (1.55%) | | 333 (1.05%) | | | 366 (1.13%) | |  |
|  | Diabetes | 1184 (20.53%) | | 1166 (20.46%) | | 1821 (17.02%) | | | 1908 (17.72%) | | 1104 (19.25%) | | | 1166 (20.46%) | | 1924 (17.94%) | | | 1907 (17.72%) | | 11607 (20.47%) | | | 11033 (19.66%) | | 5724 (18.05%) | | | 6350 (19.67%) | |  |
|  | Diabetes with end-organ damage | 61 (1.06%) | | 66 (1.16%) | | 148 (1.38%) | | | 156 (1.45%) | | 56 (0.98%) | | | 66 (1.16%) | | 135 (1.26%) | | | 156 (1.45%) | | 650 (1.15%) | | | 592 (1.05%) | | 593 (1.87%) | | | 629 (1.95%) | |  |
|  | Hemiplegia | 512 (8.88%) | | 522 (9.16%) | | 783 (7.32%) | | | 915 (8.5%) | | 512 (8.93%) | | | 522 (9.16%) | | 903 (8.42%) | | | 911 (8.47%) | | 2369 (4.18%) | | | 2240 (3.99%) | | 1234 (3.89%) | | | 1229 (3.81%) | |  |
|  | Moderate or severe renal disease | 107 (1.86%) | | 139 (2.44%) | | 591 (5.52%) | | | 590 (5.48%) | | 74 (1.29%) | | | 139 (2.44%) | | 613 (5.72%) | | | 590 (5.48%) | | 1799 (3.17%) | | | 1534 (2.73%) | | 2873 (9.06%) | | | 3140 (9.72%) | |  |
|  | Any tumor (except for malignant neoplasm of skin) | 383 (6.64%) | | 423 (7.42%) | | 855 (7.99%) | | | 934 (8.68%) | | 374 (6.52%) | | | 423 (7.42%) | | 889 (8.29%) | | | 933 (8.67%) | | 3970 (7%) | | | 4105 (7.31%) | | 2493 (7.86%) | | | 2624 (8.13%) | |  |
|  | Metastatic solid tumor | 68 (1.18%) | | 67 (1.18%) | | 193 (1.8%) | | | 214 (1.99%) | | 75 (1.31%) | | | 67 (1.18%) | | 178 (1.66%) | | | 214 (1.99%) | | 813 (1.43%) | | | 880 (1.57%) | | 533 (1.68%) | | | 520 (1.61%) | |  |
|  | HIV/ AIDS | 6 (0.1%) | | 5 (0.09%) | | 7 (0.07%) | | | 7 (0.07%) | | 3 (0.05%) | | | 5 (0.09%) | | 5 (0.05%) | | | 7 (0.07%) | | 49 (0.09%) | | | 48 (0.09%) | | 15 (0.05%) | | | 25 (0.08%) | |  |
|  | Moderate or severe liver disease | 9 (0.16%) | | 12 (0.21%) | | 29 (0.27%) | | | 43 (0.4%) | | 20 (0.35%) | | | 12 (0.21%) | | 35 (0.33%) | | | 42 (0.39%) | | 161 (0.28%) | | | 175 (0.31%) | | 73 (0.23%) | | | 62 (0.19%) | |  |
| **CHA_2_DS_2_-VASc score** | Mean (SD) | 3.2 [1.6] | | 3.2 [1.5] | | 4 [1.4] | | | 4.1 [1.4] | | 3.2 [1.6] | | | 3.2 [1.5] | | 4 [1.4] | | | 4.1 [1.4] | | 3.3 [1.5] | | | 3.3 [1.5] | | 4.1 [1.3] | | | 4.1 [1.3] | |  |
|  | 0 | 238 (4.13%) | | 197 (3.46%) | | 64 (0.6%) | | | 59 (0.55%) | | 237 (4.13%) | | | 197 (3.46%) | | 61 (0.57%) | | | 59 (0.55%) | | 1634 (2.88%) | | | 2072 (3.69%) | | 119 (0.38%) | | | 139 (0.43%) | |  |
|  | 1 | 580 (10.06%) | | 579 (10.16%) | | 203 (1.9%) | | | 193 (1.79%) | | 560 (9.76%) | | | 579 (10.16%) | | 268 (2.5%) | | | 193 (1.79%) | | 4677 (8.25%) | | | 4859 (8.66%) | | 462 (1.46%) | | | 640 (1.98%) | |  |
|  | 2–3 | 2654 (46.0%) | | 2595 (45.53%) | | 3534 (33.03%) | | | 3393 (31.52%) | | 2601 (45.35%) | | | 2595 (45.53%) | | 3552 (33.12%) | | | 3392 (31.53%) | | 25653 (45.24%) | | | 24568 (43.77%) | | 9304 (29.34%) | | | 9843 (30.49%) | |  |
|  | ≥4 | 2296 (39.82%) | | 2328 (40.85%) | | 6897 (64.47%) | | | 7120 (66.14%) | | 2338 (40.76%) | | | 2328 (40.85%) | | 6842 (63.81%) | | | 7116 (66.13%) | | 24736 (43.63%) | | | 24736 (43.63%) | | 21829 (68.83%) | | | 21667 (67.1%) | |  |
| **Concomitant treatment** | Antiplatelets | 2779 (48.2%) | | 2831 (49.68%) | | 5053 (47.23%) | | | 4999 (46.44%) | | 2706 (47.18%) | | | 2831 (49.68%) | | 5274 (49.18%) | | | 4998 (46.45%) | | 27824 (49.07%) | | | 26720 (47.61%) | | 15843 (49.96%) | | | 16894 (52.32%) | |  |
|  | Aromatase inhibitors | 28 (0.49%) | | 36 (0.63%) | | 75 (0.7%) | | | 69 (0.64%) | | 32 (0.56%) | | | 36 (0.63%) | | 95 (0.89%) | | | 69 (0.64%) | | 348 (0.61%) | | | 355 (0.63%) | | 262 (0.83%) | | | 250 (0.77%) | |  |
|  | NSAIDs | 1010 (17.52%) | | 967 (16.97%) | | 898 (8.39%) | | | 909 (8.44%) | | 904 (15.76%) | | | 967 (16.97%) | | 910 (8.49%) | | | 909 (8.45%) | | 8465 (14.93%) | | | 8534 (15.21%) | | 2440 (7.69%) | | | 2590 (8.02%) | |  |
|  | H2-receptor antagonists | 24 (0.42%) | | 26 (0.46%) | | 45 (0.42%) | | | 44 (0.41%) | | 16 (0.28%) | | | 26 (0.46%) | | 37 (0.35%) | | | 44 (0.41%) | | 219 (0.39%) | | | 223 (0.4%) | | 130 (0.41%) | | | 135 (0.42%) | |  |
|  | Prostaglandins | 211 (3.66%) | | 166 (2.91%) | | 214 (2%) | | | 191 (1.77%) | | 155 (2.7%) | | | 166 (2.91%) | | 170 (1.59%) | | | 191 (1.78%) | | 1858 (3.28%) | | | 1702 (3.03%) | | 676 (2.13%) | | | 619 (1.92%) | |  |
|  | Proton pump inhibitors | 2406 (41.73%) | | 2369 (41.57%) | | 4946 (46.23%) | | | 5063 (47.03%) | | 2351 (40.99%) | | | 2369 (41.57%) | | 5006 (46.68%) | | | 5059 (47.02%) | | 23630 (41.68%) | | | 23025 (41.02%) | | 14830 (46.76%) | | | 15490 (47.97%) | |  |
|  | Anticonvulsant strong inhibitor of hepatic enzymes | 28 (0.49%) | | 31 (0.54%) | | 68 (0.64%) | | | 62 (0.58%) | | 26 (0.45%) | | | 31 (0.54%) | | 52 (0.48%) | | | 62 (0.58%) | | 306 (0.54%) | | | 335 (0.6%) | | 188 (0.59%) | | | 186 (0.58%) | |  |
|  | HIV protease inhibitors | 35 (0.61%) | | 28 (0.49%) | | 21 (0.2%) | | | 27 (0.25%) | | 25 (0.44%) | | | 28 (0.49%) | | 22 (0.21%) | | | 27 (0.25%) | | 224 (0.4%) | | | 262 (0.47%) | | 102 (0.32%) | | | 87 (0.27%) | |  |
|  | Strong inhibitors of both CYP3A4 and P-gp | 108 (1.87%) | | 105 (1.84%) | | 125 (1.17%) | | | 153 (1.42%) | | 87 (1.52%) | | | 105 (1.84%) | | 116 (1.08%) | | | 152 (1.41%) | | 979 (1.73%) | | | 964 (1.72%) | | 391 (1.23%) | | | 423 (1.31%) | |  |
|  | Statins | 1004 (17.41%) | | 961 (16.86%) | | 1684 (15.74%) | | | 1811 (16.82%) | | 931 (16.23%) | | | 961 (16.86%) | | 1742 (16.25%) | | | 1810 (16.82%) | | 9790 (17.27%) | | | 9431 (16.8%) | | 4765 (15.02%) | | | 5220 (16.17%) | |  |
|  | Selective estrogen receptor modulators | 9 (0.16%) | | 11 (0.19%) | | 30 (0.28%) | | | 21 (0.2%) | | 5 (0.09%) | | | 11 (0.19%) | | 23 (0.21%) | | | 21 (0.2%) | | 100 (0.18%) | | | 108 (0.19%) | | 64 (0.2%) | | | 58 (0.18%) | |  |
|  | Selective serotonin reuptake inhibitors | 357 (6.19%) | | 383 (6.72%) | | 869 (8.12%) | | | 924 (8.58%) | | 344 (6%) | | | 383 (6.72%) | | 886 (8.26%) | | | 924 (8.59%) | | 3519 (6.21%) | | | 3713 (6.62%) | | 2649 (8.35%) | | | 2689 (8.33%) | |  |
|  | Hormones | 278 (4.82%) | | 245 (4.3%) | | 339 (3.17%) | | | 301 (2.8%) | | 215 (3.75%) | | | 245 (4.3%) | | 312 (2.91%) | | | 301 (2.8%) | | 2538 (4.48%) | | | 2426 (4.32%) | | 1009 (3.18%) | | | 994 (3.08%) | |  |
|  | Erythropoesis stimulating agents | 7 (0.12%) | | 11 (0.19%) | | 40 (0.37%) | | | 35 (0.33%) | | 15 (0.26%) | | | 11 (0.19%) | | 41 (0.38%) | | | 35 (0.33%) | | 110 (0.19%) | | | 157 (0.28%) | | 171 (0.54%) | | | 165 (0.51%) | |  |
|  | Beta blockers | 3379 (58.6%) | | 3298 (57.87%) | | 5786 (54.08%) | | | 5927 (55.06%) | | 3211 (55.98%) | | | 3298 (57.87%) | | 5979 (55.76%) | | | 5923 (55.05%) | | 33122 (58.42%) | | | 32117 (57.22%) | | 17543 (55.32%) | | | 18412 (57.02%) | |  |
|  | Antiarrhythmic agents | 3621 (62.8%) | | 3631 (63.71%) | | 6467 (60.45%) | | | 6299 (58.51%) | | 3641 (63.48%) | | | 3631 (63.71%) | | 6615 (61.69%) | | | 6297 (58.52%) | | 35246 (62.16%) | | | 34682 (61.79%) | | 18975 (59.83%) | | | 19443 (60.22%) | |  |

Scores (Charlson Comorbidity Index, HAS-BLED and CHA_2_DS_2_-VASc) were not included in the PS modelling as their components are singularly included, but were used as indicator for evaluating the fitness of the matching. AIDS, acquired immunodeficiency syndrome; CYP3A4, cytochrome P450 3A4; DOAC, direct oral anticoagulant; GIB, gastrointestinal bleed; HIV, human immunodeficiency virus; LTR, long-term recurrence; NSAID, nonsteroidal anti-inflammatory drug; P-gp, P-glycoprotein; PS, propensity score; SD, standard deviation; VKA, vitamin K antagonist.
