# Supplementary material for: Comparative safety and effectiveness of oral anticoagulants in patients with non-valvular atrial fibrillation and high risk of gastrointestinal bleeding: A nationwide French cohort study
Source: PLoS One. 2024 Nov 15;19(11):e0310322. doi: 10.1371/journal.pone.0310322 (PMC11567525; doi:10.1371/journal.pone.0310322)
Supplement: S4 Table — (DOCX) [file pone.0310322.s004.docx]

**Supplementary Table 4**. Demographic and clinical characteristics prior to PS matching

| **Characteristic** | | **Overall**  **(N = 314,184)** | **VKAs**  **(n = 47,142)** | **Apixaban**  **(n = 162,150)** | **Rivaroxaban (n = 88,427)** | **Dabigatran (n = 16,465)** | |
| --- | --- | --- | --- | --- | --- | --- | --- |
| **Atrial fibrillation identification setting** | Inpatient claim with I48 code | 203,030  (64.6%) | 36,720  (77.9%) | 107,436  (66.2%) | 49,621  (56.1%) | 9,253  (56.2%) |  |
|  | LTR registration with I48 code | 28,045  (8.9%) | 2,053  (4.4%) | 14,126  (8.7%) | 9,962  (11.3%) | 1,904  (11.6%) |  |
|  | Use of anti-arrhythmic drugs | 83,109  (26.4%) | 8,369  (17.8%) | 40,588  (25.0%) | 28,844  (32.6%) | 5,308  (32.2%) |  |
| **Age at index date (years)** | | 79.0 (10.5) | 81.5 (10.2) | 79.8 (10.1) | 76.6 (10.8) | 77.8 (10.1) |  |
| **Age groups at index date** | 18-54 years | 7818 (2.5%) | 795 (1.7%) | 3271 (2.0%) | 3360 (3.8%) | 392 (2.4%) |  |
|  | 55-64 years | 21703 (6.9%) | 2395 (5.1%) | 9769 (6.0%) | 8272 (9.4%) | 1267 (7.7%) |  |
|  | 65-74 years | 58626 (18.7%) | 6917 (14.7%) | 28414 (17.58%) | 19775 (22.4%) | 3520 (21.4%) |  |
|  | 75-79 years | 54495 (17.3%) | 6290 (13.3%) | 27567 (17.0%) | 17456 (19.7%) | 3182 (19.3%) |  |
|  | ≥80 years | 171542 (54.6%) | 30745 (65.2%) | 93129 (57.4%) | 39564 (44.7%) | 8104 (49.2%) |  |
| **Sex** | Male | 153960 (49.0%) | 22578 (47.9%) | 75714 (46.7%) | 47189 (53.4%) | 8479 (51.5%) |  |
|  | Female | 160224 (51.0%) | 24564 (52.1%) | 86436 (53.3%) | 41238 (46.6%) | 7986 (48.5%) |  |
| **GIB risk factors** | Age ≥75 years | 226037 (71.9%) | 37035 (78.6%) | 120696 (74.4%) | 57020 (64.5%) | 11286 (68.5%) |  |
|  | HAS-BLED score, mean | 2.7 (1.0) | 3.1 (1.1) | 2.7 (1.0) | 2.5 (1.0) | 2.6 (1.0) |  |
|  | 0 | 2200 (0.1%) | 108 (0.2%) | 963 (0.6%) | 1015 (1.1%) | 114 (0.7%) |  |
|  | 1 | 29780 (9.5%) | 2486 (5.3%) | 14733 (9.1%) | 10807 (12.2%) | 1754 (10.7%) |  |
|  | 2 | 96181 (30.6%) | 10852 (23.0%) | 50308 (31.0%) | 29758 (33.7%) | 5263 (32.0%) |  |
|  | ≥3 | 186023 (59.2%) | 33696 (71.5%) | 96146 (59.3%) | 46847 (53.0%) | 9334 (56.7%) |  |
|  | Prior medications | 193868 (61.7%) | 27340 (58%) | 98803 (60.9%) | 57580 (65.1%) | 10145 (61.6%) |  |
|  | Renal impairment | 18703 (6.0%) | 8058 (17.1%) | 7594 (4.7%) | 2656 (3.0%) | 395 (2.4%) |  |
|  | Prior GI condition | 25200 (8.0%) | 4265 (9.0%) | 12501 (7.7%) | 7060 (8.0%) | 1374 (8.3%) |  |
| **Number of GIB risk factors** | 1 | 114354 (36.4%) | 12546 (26.6%) | 58347 (36.0%) | 36953 (41.8%) | 6508 (39.5%) |  |
|  | 2 | 80874 (26%) | 11566 (25%) | 41752 (25.7%) | 23130 (26.2%) | 4426 (26.9%) |  |
|  | 3 | 102966 (32.8%) | 17760 (37.7%) | 54676 (33.7%) | 25555 (28.9%) | 4975 (30.2%) |  |
|  | 4 | 15119 (4.8%) | 4914 (10.4%) | 7014 (4.3%) | 2660 (3.0%) | 531 (3.2%) |  |
|  | 5 | 871 (0.3%) | 356 (0.8%) | 361 (0.2%) | 129 (0.1%) | 25 (0.2%) |  |
| **Charlson Comorbidity Index score** | 0 | 93985 (29.9%) | 7612 (16.1%) | 48044 (29.6%) | 32552 (36.8%) | 5777 (35.1%) |  |
|  | 1 or 2 | 136180 (43.3%) | 18359 (38.9%) | 72233 (44.5%) | 38617 (43.7%) | 6971 (42.3%) |  |
|  | 3 or 4 | 55291 (17.6%) | 12389 (26.3%) | 28554 (17.6%) | 11788 (13.3%) | 2560 (15.5%) |  |
|  | ≥5 | 28728 (9.1%) | 8782 (18.6%) | 13319 (8.2%) | 5470 (6.2%) | 1157 (7.0%) |  |
| **Charlson Comorbidities** | Myocardial infarction | 24275 (7.7%) | 5456 (11.6%) | 11789 (7.3%) | 6144 (6.9%) | 886 (5.4%) |  |
|  | Congestive heart failure | 96455 (30.7%) | 22185 (47.1%) | 48880 (30.1%) | 21768 (24.6%) | 3622 (22%) |  |
|  | Peripheral vascular disease | 26921 (8.6%) | 6511 (13.8%) | 13062 (8.1%) | 6238 (7.1%) | 1110 (6.7%) |  |
|  | Cerebrovascular disease | 49969 (15.9%) | 8652 (18.4%) | 28370 (17.5%) | 9801 (11.1%) | 3146 (19.1%) |  |
|  | Dementia | 24361 (7.8%) | 5308 (11.3%) | 13091 (8.1%) | 5115 (5.8%) | 847 (5.1%) |  |
|  | Chronic pulmonary disease | 64956 (20.7%) | 10866 (23%) | 33041 (20.4%) | 17916 (20.3%) | 3133 (19%) |  |
|  | Connective tissue disease | 4787 (1.5%) | 865 (1.8%) | 2588 (1.6%) | 1128 (1.3%) | 206 (1.3%) |  |
|  | Ulcer disease | 3130 (1%) | 767 (1.6%) | 1531 (0.9%) | 673 (0.8%) | 159 (1%) |  |
|  | Mild liver disease | 5005 (1.6%) | 1288 (2.7%) | 2263 (1.4%) | 1236 (1.4%) | 218 (1.3%) |  |
|  | Diabetes | 63863 (20.3%) | 11844 (25.1%) | 31560 (19.5%) | 17385 (19.7%) | 3074 (18.7%) |  |
|  | Diabetes with end-organ damage | 6785 (2.2%) | 2519 (5.3%) | 2823 (1.7%) | 1221 (1.4%) | 222 (1.3%) |  |
|  | Hemiplegia | 20479 (6.5%) | 3582 (7.6%) | 11989 (7.4%) | 3470 (3.9%) | 1438 (8.7%) |  |
|  | Moderate or severe renal disease | 30513 (9.7%) | 12409 (26.3%) | 12700 (7.8%) | 4675 (5.3%) | 729 (4.4%) |  |
|  | Any tumor (including lymphoma and leukemia except for malignant neoplasm of skin) | 25824 (8.2%) | 4920 (10.4%) | 12818 (7.9%) | 6729 (7.6%) | 1357 (8.2%) |  |
|  | Metastatic solid tumor | 5113 (1.6%) | 989 (2.1%) | 2443 (1.5%) | 1400 (1.6%) | 281 (1.7%) |  |
|  | HIV/AIDS | 265 (0.1%) | 80 (0.2%) | 99 (0.1%) | 74 (0.1%) | 12 (0.1%) |  |
|  | Moderate or severe liver disease | 1106 (0.4%) | 367 (0.8%) | 447 (0.3%) | 237 (0.3%) | 55 (0.3%) |  |
| **Additional comorbidities** | Anemia and coagulation defects | 36305 (11.6%) | 9768 (20.7%) | 17785 (11%) | 7340 (8.3%) | 1412 (8.6%) |  |
|  | Baseline prior bleed | 49971 (15.9%) | 12437 (26.4%) | 24860 (15.3%) | 10461 (11.8%) | 2213 (13.4%) |  |
|  | Thrombocytopenia | 3395 (1.1%) | 900 (1.9%) | 1569 (1%) | 764 (0.9%) | 162 (1%) |  |
|  | Atherosclerotic disease | 18367 (5.8%) | 4533 (9.6%) | 8992 (5.5%) | 4143 (4.7%) | 699 (4.2%) |  |
|  | Ischemic heart disease or myocardial infarction | 23230 (7.4%) | 5283 (11.2%) | 11256 (6.9%) | 5865 (6.6%) | 826 (5%) |  |
|  | History of stroke or transient ischemic attack | 38206 (12.2%) | 6332 (13.4%) | 22394 (13.8%) | 6929 (7.8%) | 2551 (15.5%) |  |
|  | Vascular disease | 60815 (19.4%) | 13483 (28.6%) | 30739 (19%) | 14158 (16%) | 2435 (14.8%) |  |
|  | Heart failure | 82566 (26.3%) | 19522 (41.4%) | 41872 (25.8%) | 18189 (20.6%) | 2983 (18.1%) |  |
|  | Dyspepsia or stomach discomfort | 8300 (2.6%) | 1396 (3%) | 4249 (2.6%) | 2257 (2.6%) | 398 (2.4%) |  |
|  | Coronary artery disease | 59884 (19.1%) | 12850 (27.3%) | 29575 (18.2%) | 15038 (17%) | 2421 (14.7%) |  |
|  | Hypertension | 260613 (82.9%) | 42000 (89.1%) | 135041 (83.3%) | 70252 (79.4%) | 13320 (80.9%) |  |
|  | Obesity | 36274 (11.5%) | 7152 (15.2%) | 17560 (10.8%) | 9804 (11.1%) | 1758 (10.7%) |  |
|  | Peptic ulcer disease | 3130 (1%) | 767 (1.6%) | 1531 (0.9%) | 673 (0.8%) | 159 (1%) |  |
|  | Liver disease | 5334 (1.7%) | 1367 (2.9%) | 2419 (1.5%) | 1321 (1.5%) | 227 (1.4%) |  |
|  | Chronic kidney disease | 28534 (9.1%) | 11685 (24.8%) | 11887 (7.3%) | 4293 (4.9%) | 669 (4.1%) |  |
|  | *Helicobacter pylori* | 1559 (0.5%) | 197 (0.4%) | 780 (0.5%) | 489 (0.6%) | 93 (0.6%) |  |
|  | Chronic obstructive pulmonary disease | 1117 (0.4%) | 275 (0.6%) | 520 (0.3%) | 265 (0.3%) | 57 (0.3%) |  |
|  | Diverticulosis | 13841 (4.4%) | 1868 (4%) | 6966 (4.3%) | 4248 (4.8%) | 759 (4.6%) |  |
|  | SE | 5124 (1.6%) | 1471 (3.1%) | 2332 (1.4%) | 1138 (1.3%) | 183 (1.1%) |  |
|  | Angiodysplasia | 307 (0.1%) | 70 (0.1%) | 156 (0.1%) | 60 (0.1%) | 21 (0.1%) |  |
|  | GI cancer | 4463 (1.4%) | 863 (1.8%) | 2177 (1.3%) | 1161 (1.3%) | 262 (1.6%) |  |
|  | GI lesions | 469 (0.1%) | 90 (0.2%) | 246 (0.2%) | 106 (0.1%) | 27 (0.2%) |  |
| **CHA_2_DS_2_-VASc score** | Mean (SD) | 3.9 (1.5) | 4.4 (1.4) | 4.0 (1.5) | 3.6 (1.5) | 3.8 (1.5) | |
|  | 0 | 4486 (1.4%) | 193 (0.4%) | 1817 (1.1%) | 2220 (2.5%) | 256 (1.6%) |  |
|  | 1 | 12649 (4.0%) | 843 (1.8%) | 5533 (3.4%) | 5501 (6.2%) | 772 (4.7%) |  |
|  | 2–3 | 100911 (32.1%) | 10544 (22.4%) | 49967 (30.8%) | 34412 (38.9%) | 5988 (36.4%) |  |
|  | ≥4 | 196138 (62.4%) | 35562 (75.4%) | 104833 (64.7%) | 46294 (52.4%) | 9449 (57.4%) |  |
| **Concomitant treatment** | Antiplatelets | 151759 (48.3%) | 23268 (49.4%) | 77045 (47.5%) | 43616 (49.3%) | 7830 (47.6%) |  |
|  | Aromatase inhibitors | 2202 (0.7%) | 350 (0.7%) | 1142 (0.7%) | 605 (0.7%) | 105 (0.6%) |  |
|  | NSAIDs | 33395 (10.6%) | 2666 (5.7%) | 17723 (10.9%) | 11130 (12.6%) | 1876 (11.4%) |  |
|  | Corticosteroids | 10632 (3.4%) | 769 (1.6%) | 6535 (4%) | 2853 (3.2%) | 475 (2.9%) |  |
|  | H2-receptor antagonists | 1291 (0.4%) | 222 (0.5%) | 641 (0.4%) | 358 (0.4%) | 70 (0.4%) |  |
|  | Prostaglandins | 8972 (2.9%) | 630 (1.3%) | 5664 (3.5%) | 2321 (2.6%) | 357 (2.2%) |  |
|  | Proton pump inhibitors | 147078 (46.8%) | 25644 (54.4%) | 75487 (46.6%) | 38515 (43.6%) | 7432 (45.1%) |  |
|  | Anticonvulsant strong inhibitor of hepatic enzymes | 1907 (0.6%) | 386 (0.8%) | 907 (0.6%) | 521 (0.6%) | 93 (0.6%) |  |
|  | HIV protease inhibitors | 1596 (0.5%) | 142 (0.3%) | 1050 (0.6%) | 349 (0.4%) | 55 (0.3%) |  |
|  | Strong inhibitors of both CYP3A4 and P-gp | 5951 (1.9%) | 548 (1.2%) | 3758 (2.3%) | 1387 (1.6%) | 258 (1.6%) |  |
|  | Statins | 51494 (16.4%) | 7667 (16.3%) | 26403 (16.3%) | 14652 (16.6%) | 2772 (16.8%) |  |
|  | Selective estrogen receptor modulators | 564 (0.2%) | 82 (0.2%) | 284 (0.2%) | 166 (0.2%) | 32 (0.2%) |  |
|  | Selective serotonin reuptake inhibitors | 26112 (8.3%) | 4410 (9.4%) | 13991 (8.6%) | 6404 (7.2%) | 1307 (7.9%) |  |
|  | Hormones | 12293 (3.9%) | 933 (2%) | 7394 (4.6%) | 3420 (3.9%) | 546 (3.3%) |  |
|  | Erythropoesis stimulating agents | 2323 (0.7%) | 1132 (2.4%) | 823 (0.5%) | 322 (0.4%) | 46 (0.3%) |  |
|  | Beta blockers | 185590 (59.1%) | 29124 (61.8%) | 96710 (59.6%) | 50530 (57.1%) | 9226 (56%) |  |
|  | Antiarrhythmic agents | 173851 (55.3%) | 22416 (47.5%) | 87367 (53.9%) | 54137 (61.2%) | 9931 (60.3%) |  |
|  | Angiotensin-converting-enzyme inhibitors/angiotensin II receptor blockers | 158196 (50.4%) | 23745 (50.4%) | 82141 (50.7%) | 43991 (49.7%) | 8319 (50.5%) |  |
| **Dosage at index date** | Standard dose | 152831 (48.6%) |  | 90995 (56.1%) | 56137 (63.5%) | 5699 (34.6%) | |
|  | Reduce dose | 114211 (36.4%) |  | 71155 (43.9%) | 32290 (36.5%) | 10766 (65.4%) | |

*Standard dose: Apixaban 5 mg, Rivaroxaban 20 mg, Dabigatran 150 mg; Reduced dose: Apixaban 2.5 mg, Rivaroxaban 15 mg, Dabigatran 110 mg

AIDS, acquired immunodeficiency syndrome; CYP3A4, cytochrome P450 3A4; DOAC, direct oral anticoagulant; GIB, gastrointestinal bleed; HIV, human immunodeficiency virus; LTR, long-term recurrence; NSAID, nonsteroidal anti-inflammatory drug; P-gp, P-glycoprotein; PS, propensity score; SD, standard deviation; VKA, vitamin K antagonist.
