# Supplementary material for: Comparative safety and effectiveness of oral anticoagulants in patients with non-valvular atrial fibrillation and high risk of gastrointestinal bleeding: A nationwide French cohort study
Source: PLoS One. 2024 Nov 15;19(11):e0310322. doi: 10.1371/journal.pone.0310322 (PMC11567525; doi:10.1371/journal.pone.0310322)
Supplement: S5 Table — (DOCX) [file pone.0310322.s005.docx]

**Supplementary Table 5**. Outcome rates by cohort prior to PS matching.

|  |  | **Overall**  **(N = 314,184)** | **VKAs**  **(n = 47,142)** | **Apixaban**  **(n = 162,150)** | **Rivaroxaban (n = 88,427)** | **Dabigatran**  **(n = 16,465)** |
| --- | --- | --- | --- | --- | --- | --- |
| **Bleeding leading to hospitalization (primary diagnosis only)** | |  |  |  |  |  |
|  | N (%) | 10702 (3.4%) | 2518 (5.3%) | 4439 (2.7%) | 3248 (3.7%) | 497 (3%) |
|  | Time at risk, person-years | 333640.8 | 40986 | 180300.6 | 94107.1 | 18247.1 |
|  | Incidence rate (95% CI), per 100 person year † | 3.21 (3.15, 3.27) | 6.14 (5.91, 6.39) | 2.46 (2.39, 2.54) | 3.45 (3.33, 3.57) | 2.72 (2.49, 2.97) |
|  | Time to event (days), median (IQR)** | 192.0 (58.0 – 456.0) | 157.0 (47.0 – 433.0) | 204.0 (67.0 – 476.0) | 198.0 (56.0 – 444.0) | 197.0 (59.0 – 475.0) |
| **ICH** | |  |  |  |  |  |
|  | N (%) | 2396 (0.8%) | 617 (1.3%) | 1139 (0.7%) | 579 (0.7%) | 61 (0.4%) |
|  | Time at risk, person-years | 339547.7 | 42386 | 182754.2 | 95921.8 | 18485.7 |
|  | Incidence rate (95% CI), per 100 person year † | 0.71 (0.68, 0.73) | 1.46 (1.35, 1.58) | 0.62 (0.59, 0.66) | 0.6 (0.56, 0.65) | 0.33 (0.26, 0.42) |
|  | Time to event (days), median (IQR)** | 228.0 (82.0 – 483.0) | 204.0 (70.0 – 466.0) | 225.0 (82.0 – 484.0) | 264.0 (98.0 – 510.0) | 207.0 (79.0 – 436.0) |
| **GIB** | |  |  |  |  |  |
|  | N (%) | 4117 (1.3%) | 921 (2%) | 1564 (1%) | 1353 (1.5%) | 279 (1.7%) |
|  | Time at risk, person-years | 337496.7 | 41972.2 | 181976 | 95189.7 | 18358.9 |
|  | Incidence rate (95% CI), per 100 person year † | 1.22 (1.18, 1.26) | 2.19 (2.06, 2.34) | 0.86 (0.82, 0.9) | 1.42 (1.35, 1.5) | 1.52 (1.35, 1.71) |
|  | Time to event (days), median (IQR)** | 187.0 (51.0 – 461.0) | 168.0 (39.0 – 449.0) | 203.0 (62.0 – 472.5) | 182.0 (49.0 – 444.0) | 192.0 (43.0 – 515.0) |
| **Other bleeding** | |  |  |  |  |  |
|  | N (%) | 4577 (1.5%) | 1105 (2.3%) | 1857 (1.1%) | 1437 (1.6%) | 178 (1.1%) |
|  | Time at risk, person-years | 336906.9 | 41728 | 181709.7 | 95072 | 18397.1 |
|  | Incidence rate (95% CI), per 100 person year † | 1.36 (1.32, 1.4) | 2.65 (2.5, 2.81) | 1.02 (0.98, 1.07) | 1.51 (1.44, 1.59) | 0.97 (0.84, 1.12) |
|  | Time to event (days), median (IQR)** | 190.0 (55.0 – 455.0) | 145.0 (44.0 – 413.0) | 203.0 (63.0 – 488.0) | 197.0 (52.0 – 438.0) | 209.5 (86.0 – 447.0) |
| **Stroke (ischemic or hemorrhagic) or SE (primary diagnosis only)** | |  |  |  |  |  |
|  | N (%) | 6467 (2.1%) | 1348 (2.9%) | 3195 (2%) | 1608 (1.8%) | 316 (1.9%) |
|  | Time at risk, person-years | 336599.8 | 41919.3 | 181096.4 | 95250.7 | 18333.4 |
|  | Incidence rate (95% CI), per 100 person year † | 1.92 (1.88, 1.97) | 3.22 (3.05, 3.39) | 1.76 (1.7, 1.83) | 1.69 (1.61, 1.77) | 1.72 (1.54, 1.92) |
|  | Time to event (days), median (IQR)** | 193.0 (64.0 – 457.0) | 182.0 (59.0 – 442.5) | 185.0 (62.0 – 445.0) | 216.0 (71.0 – 489.5) | 201.5 (71.0 – 429.0) |
| **Stroke (ischemic or hemorrhagic)** | |  |  |  |  |  |
|  | N (%) | 4748 (1.5%) | 976 (2.1%) | 2355 (1.5%) | 1175 (1.3%) | 242 (1.5%) |
|  | Time at risk, person-years | 338039.5 | 42173.8 | 181852.5 | 95607.9 | 18405.4 |
|  | Incidence rate (95% CI), per 100 person year † | 1.4 (1.37, 1.45) | 2.31 (2.17, 2.46) | 1.3 (1.24, 1.35) | 1.23 (1.16, 1.3) | 1.31 (1.16, 1.49) |
|  | Time to event (days), median (IQR)** | 196.0 (63.0 – 467.0) | 174.0 (53.5 – 440.0) | 191.0 (62.0 – 467.0) | 217.0 (73.0 – 509.0) | 207.5 (70.0 – 409.0) |
| **Ischemic stroke** | |  |  |  |  |  |
|  | N (%) | 3511 (1.1%) | 643 (1.4%) | 1805 (1.1%) | 860 (1%) | 203 (1.2%) |
|  | Time at risk, person-years | 338303.5 | 42264.9 | 181973.5 | 95651 | 18414 |
|  | Incidence rate (95% CI), per 100 person year † | 1.04 (1, 1.07) | 1.52 (1.41, 1.64) | 0.99 (0.95, 1.04) | 0.9 (0.84, 0.96) | 1.1 (0.96, 1.26) |
|  | Time to event (days), median (IQR)** | 182.0 (55.0 – 462.0) | 163.0 (48.0 – 449.0) | 177.0 (55.0 – 439.0) | 206.5 (64.0 – 519.0) | 189.0 (68.0 – 409.0) |
| **Hemorrhagic stroke** | |  |  |  |  |  |
|  | N (%) | 1304 (0.4%) | 350 (0.7%) | 582 (0.4%) | 329 (0.4%) | 43 (0.3%) |
|  | Time at risk, person-years | 339950.9 | 42480.7 | 182961.7 | 96016.2 | 18492.3 |
|  | Incidence rate (95% CI), per 100 person year † | 0.38 (0.36, 0.4) | 0.82 (0.74, 0.91) | 0.32 (0.29, 0.35) | 0.34 (0.31, 0.38) | 0.23 (0.17, 0.31) |
|  | Time to event (days), median (IQR)** | 234.0 (89.0 – 482.5) | 203.5 (74.0 – 438.0) | 240.5 (95.0 – 512.0) | 266.0 (97.0 – 504.0) | 210.0 (95.0 – 372.0) |
| **SE** | |  |  |  |  |  |
|  | N (%) | 1761 (0.6%) | 385 (0.8%) | 858 (0.5%) | 443 (0.5%) | 75 (0.5%) |
|  | Time at risk, person-years | 338782.3 | 42318.4 | 182336.5 | 95696.6 | 18430.7 |
|  | Incidence rate (95% CI), per 100 person year † | 0.52 (0.5, 0.54) | 0.91 (0.82, 1.01) | 0.47 (0.44, 0.5) | 0.46 (0.42, 0.51) | 0.41 (0.32, 0.51) |
|  | Time to event (days), median (IQR)** | 192.0 (70.0 – 432.0) | 210.0 (86.0 – 470.0) | 175.0 (67.0 – 413.0) | 202.0 (64.0 – 464.0) | 198.0 (77.0 – 497.0) |

**Time to event is estimated among patients with the event. †: measured over the overall time-at-risk for each event (i.e. time-at-risk from index-date to time to event or censoring due to treatment non-persistence, CKD-5, dialysis, pregnancy, death, or end of the study).

CI, confidence interval; GIB, gastrointestinal bleeding; ICH, intracranial hemorrhage; IQR, interquartile range; SD, standard deviation; SE, systemic embolism; VKA, vitamin K antagonist.
