# Supplementary material for: Comparative safety and effectiveness of oral anticoagulants in patients with non-valvular atrial fibrillation and high risk of gastrointestinal bleeding: A nationwide French cohort study
Source: PLoS One. 2024 Nov 15;19(11):e0310322. doi: 10.1371/journal.pone.0310322 (PMC11567525; doi:10.1371/journal.pone.0310322)
Supplement: S6 Table — (DOCX) [file pone.0310322.s006.docx]

**Supplementary Table 6.** Estimated relative acceleration factors and 95% CI from the AFT analysis (PS matched population)

|  | **Apixaban vs VKAs**  **(n = 45,124)** | **Dabigatran vs VKAs (n = 16,415)** | **Rivaroxaban vs VKAs (n = 38,737)** | **Apixaban vs dabigatran**  **(n = 16,464)** | **Dabigatran vs rivaroxaban**  **(n = 16,459)** | **Apixaban vs rivaroxaban**  **(n = 88,414)** |
| --- | --- | --- | --- | --- | --- | --- |
| **Major bleed** | 0.418 (0.384–0.455)  *p*<0.0001 | 0.498 (0.432–0.575)  *p*<0.0001 | 0.727 (0.669–0.791)  *p*<0.0001 | 0.635 (0.527–0.764)  *p*<0.0001 | 0.682 (0.578–0.806)  *p*<0.0001 | 0.518 (0.48–0.559)  *p*<0.0001 |
| **GIB** | 0.423 (0.365–0.489)  *p*<0.0001 | 0.951 (0.759–1.193)  *p*=0.666 | 0.911 (0.793–1.046)  *p*=0.186 | 0.341 (0.253–0.459)  *p*<0.0001 | 1.082 (0.843–1.39)  *p*=0.535 | 0.416 (0.366–0.472)  *p*<0.0001 |
| **ICH** | 0.456 (0.39–0.534)  *p*<0.0001 | 0.26 (0.195–0.346)  *p*<0.0001 | 0.533 (0.455–0.624)  *p*<0.0001 | 1.627 (1.094–2.421)  *p*=0.016 | 0.459 (0.313–0.673)  *p*<0.0001 | 0.886 (0.77–1.019)  *p*=0.089 |
| **Other bleed** | 0.375 (0.327–0.431)  *p*<0.0001 | 0.353 (0.274–0.455)  *p*<0.0001 | 0.722 (0.626–0.832)  *p*<0.0001 | 0.778 (0.587–1.031)  *p*=0.080 | 0.484 (0.377–0.623)  *p*<0.0001 | 0.479 (0.426–0.539)  *p*<0.0001 |
| **Stroke/SE** | 0.674 (0.609–0.746)  *p*<0.0001 | 0.59 (0.489–0.711)  *p*<0.0001 | 0.69 (0.619–0.77)  *p*<0.0001 | 0.892 (0.714–1.114)  *p*=0.312 | 0.904 (0.735–1.111)  *p*=0.338 | 0.874 (0.801–0.955)  *p*=0.003 |
| **SE** | 0.753 (0.621–0.915)  *p*=0.004 | 0.631 (0.428–0.931)  *p*=0.020 | 0.748 (0.603–0.927)  *p*=0.008 | 0.927 (0.616–1.395)  *p*=0.717 | 0.805 (0.518–1.25)  *p*=0.333 | 0.961 (0.804–1.149)  *p*=0.666 |
| **Stroke (ischemic or hemorrhagic)** | 0.644 (0.57–0.728)  *p*<0.0001 | 0.573 (0.463–0.709)  *p*<0.0001 | 0.67 (0.588–0.764)  *p*<0.0001 | 0.885 (0.682–1.15)  *p*=0.361 | 0.922 (0.727–1.169)  *p*=0.501 | 0.841 (0.758–0.932)  *p*<0.001 |
| **Ischemic stroke** | 0.799 (0.689–0.928)  *p*=0.003 | 0.772 (0.599–0.996)  *p*=0.046 | 0.773 (0.657–0.91)  *p*=0.002 | 0.797 (0.587–1.083)  *p*=0.148 | 1.053 (0.796–1.393)  *p*=0.718 | 0.87 (0.768–0.986)  *p*=0.029 |
| **Hemorrhagic stroke** | 0.38 (0.304–0.475)  *p*<0.0001 | 0.273 (0.181–0.41)  *p*<0.0001 | 0.482 (0.384–0.604)  *p*<0.0001 | 1.192 (0.733–1.936)  *p*=0.479 | 0.69 (0.467–1.019)  *p*=0.062 | 0.782 (0.65–0.94)  *p*=0.009 |

AFT, accelerated failure time; CI, confidence interval; GIB, gastrointestinal bleeding; ICH, intracranial hemorrhage; PS, propensity score; SE, systemic embolism; VKA, vitamin K antagonist.
