# Supplementary material for: Comparative safety and effectiveness of oral anticoagulants in patients with non-valvular atrial fibrillation and high risk of gastrointestinal bleeding: A nationwide French cohort study
Source: PLoS One. 2024 Nov 15;19(11):e0310322. doi: 10.1371/journal.pone.0310322 (PMC11567525; doi:10.1371/journal.pone.0310322)

**Supplementary Figure 4**. Kaplan-Meier curves for SE with apixaban versus VKAs (A), dabigatran versus VKAs (B), and rivaroxaban versus VKAs (C), rivaroxaban versus apixaban (D), dabigatran versus apixaban (E), and dabigatran versus rivaroxaban (F)


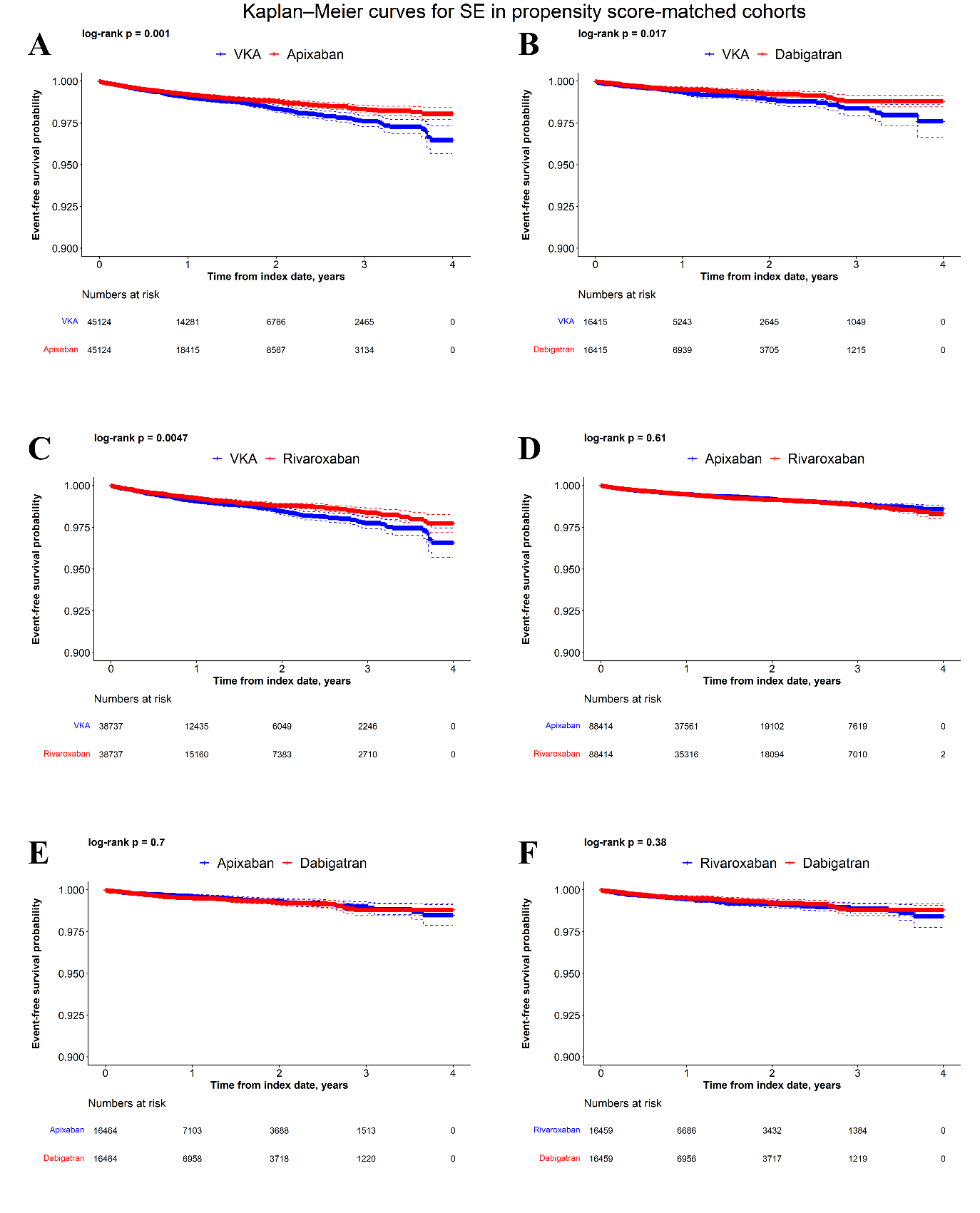

Supplement: S4 Fig — Apixaban versus VKAs (A), dabigatran versus VKAs (B), and rivaroxaban versus VKAs (C), rivaroxaban versus apixaban (D), dabigatran versus apixaban (E), and dabigatran versus rivaroxaban (F). (DOCX) [file pone.0310322.s010.docx]
