# Supplementary material for: Comparative safety and effectiveness of oral anticoagulants in patients with non-valvular atrial fibrillation and high risk of gastrointestinal bleeding: A nationwide French cohort study
Source: PLoS One. 2024 Nov 15;19(11):e0310322. doi: 10.1371/journal.pone.0310322 (PMC11567525; doi:10.1371/journal.pone.0310322)
Supplement: S7 Fig — Apixaban versus VKAs (A), dabigatran versus VKAs (B), rivaroxaban versus VKAs (C), apixaban versus dabigatran (D), dabigatran versus rivaroxaban (E), and apixaban versus rivaroxaban (F). (DOCX) [file pone.0310322.s013.docx]

**Supplementary Figure 7.** PS-matched hazard ratios for sensitivity analyses excluding probable AF: apixaban versus VKAs (A), dabigatran versus VKAs (B), rivaroxaban versus VKAs (C), apixaban versus dabigatran (D), dabigatran versus rivaroxaban (E), and apixaban versus rivaroxaban (F)

**C**

**B**

**A**

**F**

**E**

**D**
